# Checklist of beetles (Coleoptera) of Canada and Alaska. Second edition

**DOI:** 10.3897/zookeys.360.4742

**Published:** 2013-12-06

**Authors:** Yves Bousquet, Patrice Bouchard, Anthony E. Davies, Derek S. Sikes

**Affiliations:** 1 Agriculture and Agri-Food Canada, Canadian National Collection of Insects, Arachnids and Nematodes, Ottawa, Ontario, Canada K1A 0C6; 2 University of Alaska Museum, 907 Yukon Drive, Fairbanks, AK 99775-6960, USA

**Keywords:** Coleoptera, checklist, distribution, classification, adventive and Holarctic species, Alaska, Canada

## Abstract

All 8237 species-group taxa of Coleoptera known to occur in Canada and Alaska are recorded by province/territory or state, along with their author(s) and year of publication, in a classification framework. Only presence of taxa in each Canadian province or territory and Alaska is noted. Labrador is considered a distinct geographical entity. Adventive and Holarctic species-group taxa are indicated. References to pertinent identification keys are given under the corresponding supraspecific taxa in the data archive.

## Introduction

More than twenty years have passed since the publication of the *Checklist of beetles of Canada and Alaska* (Bousquet 1991). During that period many changes have been introduced into the classification of Coleoptera. For example, the scydmaenines, pselaphines, scaphidiines, and micropeplines, which were usually regarded as distinct families, are now part of the Staphylinidae, the lyctines are included in the Bostrichidae, the anobiines in the Ptinidae, the languriines in the Erotylidae, the bruchines in the Chrysomelidae, the apionines and ithycerines in the Brentidae, and the scolytines and platypodines in the Curculionidae. On the other hand, a number of family-group taxa, usually treated as subfamilies, are now considered distinct families. This is the case for the geotrupines, trogines, glaresines, ochodaeines, and the glaphyrines in the superfamily Scarabaeoidea, the silvanines, passandrines, laemophloeines, and kateretines in the Cucujoidea, and the megalopodines and orsodacnines in the Chrysomeloidea. This period also saw the publication of numerous revisions of genus-group and family-group taxa, frequently accompanied by phylogenetic analyses, which have often drastically changed our understanding of the classification of these taxa. Three volumes of the series *The Insects and Arachnids of Canada* have been issued, one dealing with the superfamily Curculionoidea (excluding the Curculionidae) (Bright 1993), one on the Histeridae (Bousquet and Laplante 2006) and one on the Curculionidae subfamily Entiminae (Bright and Bouchard 2008). Finally many Canadian provincial and territorial records have been published in the last twenty years. These represent both new introductions as well as increases in our knowledge of native species distributions largely through increased collection efforts. This is particularly true for the Maritimes, thanks to the effort of Christopher Majka and Reginald Webster. Considering the amount of new information available, we believe that a new edition of the *Checklist of beetles of Canada and Alaska* will help those interested in beetles of the Canadian fauna.

As for the first edition of the *Checklist*, this publication is intended to provide the correct names of the beetles that occur in Canada and Alaska, give a brief survey of the distribution of the species in the area covered, and lead the users to useful publications for the identification of the taxa.

## Format

The classification of family-group taxa used in this checklist follows Bouchard et al. (2011). The listing of these names is phylogenetic for the superfamilies, families, and subfamilies, starting with what is considered the most basal-grade taxa, and alphabetic for the supertribes, tribes, and subtribes. Genus-group and species-group taxa and invalid synonyms are listed alphabetically. Synonyms are not listed unless the names were considered as valid taxa in the first edition of the *Checklist*, or the taxa were described after the publication of the first edition of the *Checklist* and reported from Canada and/or neighboring states, or the names have been used in recent important works dealing with the Nearctic fauna.

The authors of all scientific names are listed along with the date of publication of the taxa. To avoid confusion, initials are included for several authors, such as H.P. Brown, W.J. Brown, H. Clark, W.E. Clark, G. Dellacasa, M. Dellacasa, D.C. Miller, K.B. Miller, W.V. Miller, P.W.J. Müller, O.F. Müller, C.R. Sahlberg, R.F. Sahlberg, J.R. Sahlberg, C. Schaeffer, J.C. Schaeffer, E.H. Smith, J.B. Smith, R.F. Smith, S.G. Smith, C.G. Thomson, J. Thomson, A. Zimmermann, C.C.A. Zimmermann as well as W. Horn, and J.E. LeConte. LeConte and Horn stand for J.L. LeConte and G. Horn respectively.

An asterisk [*] placed after a species-group name indicates that the taxon is Holarctic, a dagger [†] that it is adventive in North America, and a double dagger [‡] that its status is uncertain and the taxon could be Holarctic or adventive on this continent. Species that have been intercepted at ports of entry but are not established in Canada or Alaska are not included in the list.

References to species identification keys are listed under the corresponding supraspecific taxa in the data archive. Only references including keys useful for the determination of all or most Canadian taxa of the group treated are considered. Keys published before the 1930s are usually out-of-date and most are omitted. References to generic keys are omitted since such keys are included in the two volumes of *American Beetles* (Arnett and Thomas 2000; Arnett et al. 2002).

An abbreviation of two letters is used to indicate records for the Canadian provinces and territories and for Alaska (see section “Abbreviations”). Labrador is considered a distinct geographic entity. The distributional records are based mostly on published records and on specimens in the Canadian National Collection of Insects, Arachnids and Nematodes, in Ottawa although other Canadian and Alaskan collections were surveyed for some groups. Only records considered to be reliable are listed. The absence of a geographic entry after a species-group name indicates that the taxon was recorded from Canada but no province was specified.

### Abbreviations

AB Alberta
NB New Brunswick
ON Ontario

AK Alaska
NF Newfoundland
PE Prince Edward Island

BC British Columbia
NS Nova Scotia
QC Quebec

LB Labrador
NT Northwest Territories
SK Saskatchewan

MB Manitoba 
NU Nunavut
YT Yukon Territory

## Taxonomic coverage

All extant described species in the order Coleoptera known to occur in Canada and Alaska are included. For a summary of the number of species recorded in each beetle family see Table 1.

**Table 1.** Number of Coleoptera species for each province/territory and Alaska by family. A = adventive; H = holarctic; T = total.

	AK	YT	NT	NU	BC	AB	SK	MB	ON	QC	NB	NS	PE	LB	NF	T	H	A
Archostemata																		
Cupedidae					1				2	1						3		
Micromalthidae					1											1		
Adephaga																		
Gyrinidae	9	11	14	1	18	18	21	21	27	28	17	18	9	11	13	34	4	
Trachypachidae	1	1	1		2	1	1									2		
Rhysodidae					1				1							2		
Carabidae	254	199	217	34	504	416	349	376	532	479	333	292	174	96	178	989	95	54
Haliplidae	5	6	11		18	15	13	13	19	22	14	13	6	2	4	34	2	
Noteridae									2	2						2		
Amphizoidae	1	2			3	2										3		
Dytiscidae	103	118	119	24	173	159	134	151	163	150	105	87	38	81	79	284	52	
Polyphaga																		
Hydrophiloidea																		
Hydrophilidae	25	20	35	4	84	67	65	68	96	89	46	46	21	6	26	151	11	19
Sphaeritidae	1				1	1										1		
Histeridae	4	3	3		56	52	46	53	77	67	43	32	14		5	136	1	12
Staphylinoidea																		
Hydraenidae	3		4		21	10	9	5	6	7	3	1			1	27	1	
Ptiliidae	9		1		26	15	1	11	27	29	16	18	3		3	49	8	12
Agyrtidae	4		1		6	3			1							8		
Leiodidae	55	39	18	1	105	74	45	58	110	101	43	52	2	15	25	182	13	2
Silphidae	9	6	9	3	13	17	18	18	18	17	11	12	11	3	6	26	4	1
Staphylinidae	402	264	209	30	779	443	270	390	865	769	625	422	86	149	317	1682	106	152
Scarabaeoidea																		
Geotrupidae					1	2	3	6	8	7	4	4	1		2	12		1
Passalidae								1	1							1		
Trogidae			1		3	5	6	5	12	6	4	4	2		1	15		
Glaresidae					1	1		1								2		
Lucanidae	2	1	2		7	2	2	3	8	5	4	3	1		1	14		
Ochodaeidae					1	2	1	2	1							4		
Hybosoridae									1							1		
Glaphyridae					1											1		
Scarabaeidae	18	10	17		80	92	74	77	126	105	50	43	13	6	19	221	1	23
Scirtoidea																		
Eucinetidae	1	1	3		5	4	3	5	4	5	5	3	1			7		1
Clambidae	1				3	3	2	2	3	6	2	1			2	7		2
Scirtidae	1	1	2		9	4	3	4	15	17	14	13	7		4	25	1	1
Dascilloidea																		
Rhipiceridae									1							1		
Buprestoidea																		
Buprestidae	16	13	19		80	60	51	65	117	92	51	37	12	5	7	177	1	6
Byrrhoidea																		
Byrrhidae	15	9	13	1	16	14	13	7	11	12	7	7	4	7	7	26	3	3
Elmidae			1		14	11	4	6	17	20	11	12	4	2	5	32		
Dryopidae					2	1	1	1	3	5	3	2			1	6		1
Lutrochidae									1	1						1		
Limnichidae					1			1	3	2						3		
Heteroceridae	2	4	5		10	18	17	20	18	16	5	2	1			28		
Psephenidae									4	3	3	2				4		
Ptilodactylidae	1				2			1	2	3	1		1			4		
Elateroidea																		
Artematopodidae			1		3	1		2	3	2	1	1			1	5		
Eucnemidae	1				14	1	3	4	25	23	16	10	4		1	38		
Throscidae			2		6	1	1	1	4	4	2	3	2		1	8		
Elateridae	57	34	52	4	201	133	102	100	188	177	124	110	49	25	42	386	7	7
Lycidae	3	1	1		8	5	5	9	25	21	16	9		1	1	29	1	
Phengodidae									1							1		
Lampyridae			1		10	5	10	12	23	17	10	11	6	1	1	32		1
Cantharidae	19	14	14	4	66	39	25	33	87	79	50	34	1	6	16	154	1	3
Derodontoidea																		
Derodontidae	2				5	4			3	2	2	4			1	8		1
Nosodendridae					1				1							2		
Bostrichoidea																		
Dermestidae	9	4	6	1	33	22	21	15	28	27	12	16	11		5	49		16
Endecatomidae								1	1	1	1					1		
Bostrichidae	2	1	3		13	4	5	7	17	13	5	7	5			24	1	4
Ptinidae	11	6	2		47	23	25	28	55	56	24	36	15		12	98		19
Lymexyloidea																		
Lymexylidae						1		1	1	1	1	1			1	1		
Cleroidea																		
Trogossitidae	4	4	5		17	6	6	7	12	9	7	7	2	1	3	22	1	
Cleridae	5	4	6		23	13	13	16	34	27	15	15	7		4	52		6
Melyridae		1	3		37	15	12	7	13	14	5	3	1		1	53		2
Cucujoidea																		
Byturidae	1	1	1		1	1	1	1	1	1	1	1	1		1	1		
Sphindidae					2	2		4	5	5	4	3				6		
Biphyllidae									1	1	1					1		
Erotylidae					6	6	5	10	20	19	10	7	2			28		1
Monotomidae	5	1			13	8	8	4	18	13	9	8	2		5	27		5
Cryptophagidae	27	2	4		48	16	22	15	23	29	19	26	7	7	15	73	18	10
Silvanidae	2		1		7	6	5	7	13	14	7	8	2		3	16		6
Cucujidae	2	1	1		6	2	2	3	3	3	2	1		1	1	8	1	
Passandridae									1	1						1		
Phalacridae					3		5	4	4	3	4	3	2	1	2	8		
Laemophloeidae	1				4	2	6	7	9	11	6	8	2		2	13		3
Kateretidae	2	1	2		5	4	4	3	6	6	5	3	1		4	8	1	2
Nitidulidae	20	13	8	1	61	42	35	45	63	63	48	38	12	1	9	101	9	11
Bothrideridae					2				2							4		
Cerylonidae	1		1		3	2	2	1	6	4	3	3	1		2	8		1
Endomychidae			1		7	2	2	5	11	9	6	7	2		2	16		2
Coccinellidae	29	30	36	2	92	81	79	66	84	80	43	43	21	9	16	161	2	9
Corylophidae	2	1	2		7	4	5	4	10	10	4	8	1			16		2
Latridiidae	19	5	6		34	10	23	18	22	29	27	31	17	1	14	64	7	21
Tenebrionoidea																		
Mycetophagidae	1				6	4	7	7	10	13	8	6	2			16		2
Ciidae	6		6		19	10	7	14	16	19	7	13	2		6	29		1
Tetratomidae	3	1			9	2	2	10	13	18	9	8	2		1	20		1
Melandryidae	5	2	3		20	10	9	12	21	31	25	24	5	3	13	43	3	
Mordellidae		2	6		18	10	31	38	48	50	30	25	14			75		
Ripiphoridae					3	2	2	1	5	3	2	1				11		
Zopheridae	1		2		9	4	3	2	9	6	4	2	1		2	19		1
Tenebrionidae	8	7	5		61	43	38	43	80	70	41	39	17	2	6	141	1	12
Prostomidae					1											1		
Synchroidae								1	1	2	1	1	1			2		
Stenotrachelidae	2	1	2		5	3	3	1	4	5	3	2	2	2	2	9		
Oedemeridae	2				10	4	3	3	5	5	5	5	1		2	13		1
Meloidae	3	1	3		22	26	22	19	18	13	7	5				47		
Mycteridae					2				1	2	1	1				4		
Boridae			1		1	2	2	2	2	2	2	1				2		
Pythidae	4	3	3		5	4	4	4	4	4	5	4	2	2	1	6		
Pyrochroidae	2		1		8	5	3	4	10	12	7	5	3		1	22	1	
Salpingidae	6	1	1		9	1	1	1	3	2	2	2		1	2	15		1
Anthicidae	3	2	5		34	27	26	21	27	26	16	13	7		3	65		3
Aderidae					1		1	1	8	9	4	3	1			10		1
Scraptiidae	2	1			16	5	5	3	8	9	4	5	3		3	20		
Ischaliidae					1	1			1	1	1	1				2		
Chrysomeloidea																		
Cerambycidae	39	33	34	4	152	103	80	113	223	190	122	99	43	19	33	368	3	7
Megalopodidae	2	3	4		6	5	5	5	6	6	1	2	1			7		1
Orsodacnidae	1	1	1		1	1	1	1	1	1	1	1	1			1		
Chrysomelidae	55	49	76	1	225	265	222	277	386	322	195	168	94	19	48	598	8	55
Curculionoidea																		
Nemonychidae					6	3	1	1	2	3	2	2				8		
Anthribidae	2	2	2		5	8	8	7	13	17	11	6	1		1	20	1	1
Attelabidae			3		3	4	7	9	11	9	4	5	2		2	14		
Brentidae	4	3	3		14	21	18	20	22	22	13	11	3		1	49	1	8
Dryophthoridae					9	10	8	10	19	17	9	12	4		2	27		3
Brachyceridae	4	4	5		9	12	10	10	13	13	7	5	1	1	3	19	4	2
Curculionidae	127	93	85	8	411	295	239	228	400	386	244	218	99	15	95	823	19	106
Total	1448	1041	1115	123	3932	2863	2353	2679	4513	4127	2703	2286	899	501	1099	8237	394	628
% of total	17,6	12,7	13,6	1,5	47,8	34,8	28,6	32,6	54,8	50,2	32,9	27,8	10,9	6,1	13,4		4,8	7,6
# species in 1991 Checklist	1205	838	1031	n/a	3626	2463	1670	2351	3841	3464	1365	1320	340	373	872	7436	316	469
Difference between 1991 and 2013	243	203	84	n/a	306	400	683	328	672	663	1338	966	559	128	227	801	78	159

## Spatial coverage

This checklist includes records from all provinces and territories of Canada as well as the American state of Alaska.

## Temporal coverage

1758–2013.

## Dataset description

*Object name*: Darwin Core Archive Checklist of the beetles (Coleoptera) of Canada and Alaska. Second edition

*Character encoding*: UTF-8

*Format name*: Darwin Core Archive format

*Format version*: 1.0

*Distribution*: http://dx.doi.org/10.5886/998dbs2a

*Publication date of the data*: 2013-12-06

*Language*: English

*Licence of use*: Open Government License – Canada, version 2.0 (http://data.gc.ca/eng/open-government-licence-canada#)

*Metadata language*: English

*Date of metadata creation*: 2013-09-06

*Hierarchy level*: Dataset

## Additional information

A book which includes a formatted version of the checklist, as well as an index to supraspecific taxa, is also available from Pensoft Publishers.

## References

[B1] Abdullah M (1965) World species of the genus *Stereopalpus* and a proposed new oriental genus (Col. Anthicidae, Pedilidae). Opuscula Entomologica 30: 25–78.

[B2] Abdullah M Abdullah A (1966) *Saperda* Fabricius, 1775 = *Eutetrapha* Bates, 1884, syn. n. (Coleoptera: Cerambycidae, Lamiinae), with a catalogue, new records, colour variation and a key to the species. Proceedings of the Royal Entomological Society of London, Series B, Taxonomy 35: 87–94. doi: 10.1111/j.1365-3113.1966.tb00478.x

[B3] Ahn K-J (1996a) A review of *Diaulota* Casey (Coleoptera: Staphylinidae: Aleocharinae), with description of a new species and known larvae. The Coleopterists Bulletin 50: 270–290.

[B4] Ahn K-J (1996b) Revision of the intertidal aleocharine genus *Tarphiota* (Coleoptera: Staphylinidae). Entomological News 107: 177–185.

[B5] Ahn K-J (1997a) A review of *Liparocephalus* Mäklin (Coleoptera: Staphylinidae: Aleocharinae) with descriptions of larvae. The Pan-Pacific Entomologist 73: 79–92.

[B6] Ahn K-J (1997b) Revision and systematic position of the intertidal genus *Thinusa* Casey (Coleoptera: Staphylinidae: Aleocharinae). Entomologica Scandinavica 28: 75–81. doi: 10.1163/187631297X00178

[B7] Ahn K-J (1999) *Tarphiota densus* (Moore), a new combination and key to the species of the genus *Tarphiota* Casey (Coleoptera: Staphylinidae: Aleocharinae). Journal of the Kansas Entomological Society 71: 191193.

[B8] Ahn K-J Ashe JS (1992) Revision of the intertidal aleocharine genus *Pontomalota* Casey (Coleoptera: Staphylinidae) with a discussion of its phylogenetic relationships. Entomologica Scandinavica 23: 347–359. doi: 10.1163/187631292X00155

[B9] Ahn K-J Ashe JS (1995) Systematic position of the intertidal genus *Bryobiota* Casey and a revised phylogeny of the falagriine genera of America north of Mexico (Coleoptera: Staphylinidae: Aleocharinae). Annals of the Entomological Society of America 88: 143–154.

[B10] Ahn K-J Ashe JS (1996) Revision of the intertidal aleocharine genus *Amblopusa* Casey and description of the new genus *Paramblopusa* (Coleoptera: Staphylinidae). Journal of the New York Entomological Society 103 [1995]: 138–154.

[B11] Al Dhafe HM (2009) Revision of the North American species of *Limonius* (Coleoptera: Elateridae). Transactions of the American Entomological Society 135: 209–352. doi: 10.3157/061.135.0301

[B12] Allen RT (1972) A revision of the genus *Loxandrus* LeConte (Coleoptera: Carabidae) in North America. Entomologica Americana 46: 1–184.

[B13] Allen RT (1980) A review of the subtribe Myadi: description of a new genus and species, phylogenetic relationships, and biogeography (Coleoptera: Carabidae: Pterostichini). The Coleopterists Bulletin 34: 1–29.

[B14] Alpert GD (1994) A comparative study of the symbiotic relationships between beetles of the genus *Cremastocheilus* (Coleoptera: Scarabaeidae) and their host ants (Hymenoptera: Formicidae). Sociobiology 25: 11–276.

[B15] Anderson DM (1962) The weevil genus *Smicronyx* in America north of Mexico (Coleoptera: Curculionidae). Proceedings of the United States National Museum 113: 185–372. doi: 10.5479/si.00963801.113-3456.185

[B16] Anderson RD (1971) A revision of the Nearctic representatives of *Hygrotus* (Coleoptera: Dytiscidae). Annals of the Entomological Society of America 64: 503–512.

[B17] Anderson RD (1976) A revision of the Nearctic species of *Hygrotus* groups II and III (Coleoptera: Dytiscidae). Annals of the Entomological Society of America 69: 577–584.

[B18] Anderson RD (1983) Revision of the Nearctic species of *Hygrotus* groups IV, V and VI (Coleoptera: Dytiscidae). Annals of the Entomological Society of America 76: 173–196.

[B19] Anderson RS (1988) Systematics, phylogeny and biogeography of New World weevils traditionally of the tribe Cleonini (Coleoptera: Curculionidae; Cleoninae). Quaestiones Entomologicae 23: 431–709.

[B20] Anderson RS (1989) Revision of the subfamily Rhynchaeninae in North America (Coleoptera: Curculionidae). Transactions of the American Entomological Society 115: 207–312.

[B21] Anderson RS (2008a) A review of the genus *Cryptorhynchus* Illiger 1807 in the United States and Canada (Curculionidae: Cryptorhynchinae). The Coleopterists Bulletin 62: 168–180.

[B22] Anderson RS (2008b) A review of the genus *Eubulus* Kirsch 1869 in the United States and Canada (Curculionidae: Cryptorhynchinae). The Coleopterists Bulletin 62: 287–296. doi: 10.1649/1064.1

[B23] Anderson RS Howden AT (1994) *Tychius meliloti* Stephens new to Canada with a brief review of the species of *Tychius* Germar introduced into North America (Coleoptera: Curculionidae). The Canadian Entomologist 126: 1363–1368. doi: 10.4039/Ent1261363-6

[B24] Anderson RS Peck SB (1985) The carrion beetles of Canada and Alaska. Coleoptera: Silphidae and Agyrtidae. Agriculture Canada Publication 1778: 121 pp.

[B25] Arnett RH Jr. (1951) A revision of the Nearctic Oedemeridae (Coleoptera). American Midland Naturalist 45: 257–391. doi: 10.2307/2421732

[B26] Arnett RH Jr. (1952) A review of the Nearctic Adelocerina (Coleoptera: Elateridae, Pyrophorinae, Pyrophorini). The Wasmann Journal of Biology 10: 103–126.

[B27] Arnett RH Jr. (1953) A review of the beetle family Cephaloidae. Proceedings of the United States National Museum 103: 155–161. doi: 10.5479/si.00963801.103-3321.155

[B28] Arnett RH Jr. Thomas MC (Eds) (2000) American beetles. Volume 1. Archostemata, Myxophaga, Adephaga, Polyphaga: Staphyliniformia. CRC Press, Boca Raton, xv + 443 pp.

[B29] Arnett RH Jr. Thomas MC Skelley PE Frank JH (Eds) (2002) American beetles. Volume 2. Polyphaga: Scarabaeoidea through Curculionoidea. CRC Press, Boca Raton, xiv + 861 pp.

[B30] Ashe JS Newton AF Jr. (1993) Larvae of *Trichophya* and phylogeny of the tachyporine group of subfamilies (Coleoptera: Staphylinidae) with a review, new species and characterization of the Trichophyinae. Systematic Entomology 18: 267–286. doi: 10.1111/j.1365-3113.1993.tb00666.x

[B31] Askevold IS (1988) The genus *Neohaemonia* Székessy in North America (Coleoptera: Chrysomelidae: Donaciinae): systematics, reconstructed phylogeny, and geographic history. Transactions of the American Entomological Society 113: 360–430.

[B32] Askevold IS (1991) Classification, reconstructed phylogeny, and geographic history of the New World members of *Plateumaris* Thomson, 1859 (Coleoptera: Chrysomelidae: Donacinae). Memoirs of the Entomological Society of Canada No. 157: 175 pp.

[B33] Assing V (2008) The genus *Calodera* Mannerheim in Canada (Insecta, Coleoptera, Staphylinidae, Aleocharinae). ZooKeys (Special Issue) 2: 203–208.

[B34] Assing V Wunderle P (1995) A revision of the species of the subfamily Habrocerinae (Coleoptera: Staphylinidae) of the world. Revue Suisse de Zoologie 102: 307–359.

[B35] Atkins MD (1963) The Cupedidae of the world. The Canadian Entomologist 95: 140–162. doi: 10.4039/Ent95140-2

[B36] Ball GE (1959) A taxonomic study of the North American Licinini with notes on the Old World species of the genus *Diplocheila* Brullé (Coleoptera). Memoirs of the American Entomological Society No. 16: 258 pp.

[B37] Ball GE (1960) A review of the taxonomy of the genus *Euryderus* LeConte 1848: with notes on the North American Dapti (of authors) (Carabidae: Harpalini). The Coleopterists Bulletin 14: 44–54.

[B38] Ball GE (1966) A revision of the North American species of the subgenus *Cryobius* Chaudoir (*Pterostichus*, Carabidae, Coleoptera). Opuscula Entomologica Supplementum No. 28: 166 pp.

[B39] Ball GE Anderson JN (1962) The taxonomy and speciation of *Pseudophonus* (a subgenus of Harpalus: Harpalini: Carabidae, known to occur in North America). The Catholic University of America Press, Washington, D.C., 94 pp.

[B40] Ball GE Erwin TL (1969) A taxonomic synopsis of the tribe Loricerini (Coleoptera: Carabidae). Canadian Journal of Zoology 47: 877–907. doi: 10.1139/z69-146

[B41] Ball GE Nègre J (1972) The taxonomy of the Nearctic species of the genus *Calathus* Bonelli (Coleoptera: Carabidae: Agonini). Transactions of the American Entomological Society 98: 412–533.

[B42] Ball GE Nimmo AP (1983) Synopsis of the species of subgenus *Progaleritina* Jeannel, including reconstructed phylogeny and geographical history (Coleoptera: Carabidae: Galerita Fabricius). Transactions of the American Entomological Society 109: 295–356.

[B43] Balsbaugh EU (1966) Genus *Lexiphanes* of America north of Mexico (Coleoptera: Chrysomelidae). Proceedings of the United States National Museum 117: 655–680. doi: 10.5479/si.00963801.117-3521.655

[B44] Balsbaugh EU (1970) Review of the genus *Paria* (Coleoptera: Chrysomelidae) of North America. Annals of the Entomological Society of America 63: 453–460.

[B45] Balsbaugh EU (1983) A taxonomic revision of the genus *Phaedon* north of Mexico (Coleoptera: Chrysomelidae). North Dakota Insects, Schafer-Post Series No. 15: 73 pp.

[B46] Baranowski R (1993) Revision of the genus *Leiodes* Latreille of North and Central America (Coleoptera: Leiodidae). Entomologica Scandinavica Supplement No. 42: 149 pp.

[B47] Barber HS (1951) North American fireflies of the genus *Photuris*. Smithsonian Miscellaneous Collection No. 117: vi + 58 pp.

[B48] Barr TC Jr. (1971) The North American *Pterostichus* of the subgenus *Cylindrocharis* Casey (Coleoptera, Carabidae). American Museum Novitates No. 2445: 14 pp.

[B49] Barron JR (1971) A revision of the Trogositidae of America north of Mexico (Coleoptera: Cleroidea). Memoirs of the Entomological Society of Canada No. 75: 143 pp.

[B50] Barron JR (1996) Review of Nearctic species of *Ostoma* (Coleoptera: Cleroidea, Trogositidae). Annals of the Entomological Society of America 89: 193–202.

[B51] Beal RS Jr. (1954) Biology and taxonomy of the Nearctic species of *Trogoderma* (Coleoptera: Dermestidae). University of California Publications in Entomology 10: 35–102.

[B52] Beal RS Jr. (1967) A revisionary study of the North American dermestid beetles formerly included in the genus *Perimegatoma* (Coleoptera). Miscellaneous Publications of the Entomological Society of America 5: 281–312.

[B53] Beal RS Jr. (1970) A taxonomic and biological study of species of Attagenini (Coleoptera: Dermestidae) in the United States and Canada. Entomologica Americana 45: 141–235.

[B54] Beal RS Jr. (1985a) A taxonomic revision of the Nearctic species of *Cryptorhopalum* (Dermestidae: Coleoptera). Transactions of the American Entomological Society 111: 171–221.

[B55] Beal RS Jr. (1985b) Review of Nearctic species of *Orphilus* (Coleoptera: Desmestidae) with description of the larva of *O. subnitidus* LeConte. The Coleopterists Bulletin 39: 265–271.

[B56] Beal RS Jr. (1998) Taxonomy and biology of Nearctic species of *Anthrenus* (Coleoptera: Dermestidae). Transactions of the American Entomological Society 124: 271–332.

[B57] Becker EC (1952) The Nearctic species of *Denticollis* (Coleoptera, Elateridae). Proceedings of the Entomological Society of Washington 54: 105–114.

[B58] Becker EC (1956) Revision of the Nearctic species of *Agriotes* (Coleoptera: Elateridae). The Canadian Entomologist Supplement No. 1: 101 pp.

[B59] Becker EC (1971) Five new species of *Megapenthes* from the southwestern United States, with a key to the Nearctic species (Coleoptera: Elateridae). The Canadian Entomologist 103: 145–167. doi: 10.4039/Ent103145-2

[B60] Becker EC (1974) Revision of the Nearctic species of *Athous* (Coleoptera: Elateridae) east of the Rocky Mountains. The Canadian Entomologist 106: 711–758. doi: 10.4039/Ent106711-7

[B61] Becker EC (1979) Review of the western Nearctic species of *Athous* (Coleoptera: Elateridae) with a key to the species north of Panama. The Canadian Entomologist 111: 569–614. doi: 10.4039/Ent111569-5

[B62] Bell RT (1960) A revision of the genus *Chlaenius* Bonelli (Coleoptera: Carabidae) in North America. Miscellaneous Publications of the Entomological Society of America 1: 97–166.

[B63] Bell RT (1970) The Rhysodini of North America, Central America, and the West Indies (Coleoptera: Carabidae or Rhysodidae). Miscellaneous Publications of the Entomological Society of America 6: 289–324.

[B64] Bell RT Bell JR (1975) Two new taxa of *Clinidium* (Coleoptera: Rhysodidae or Carabidae) from the eastern U.S., with a revised key to U.S. *Clinidium*. The Coleopterists Bulletin 29: 65–68.

[B65] Bell RT Bell JR (1982) Rhysodini of the world part III. Revision of *Omoglymmius* Ganglbauer (Coleoptera: Carabidae or Rhysodidae) and substitutions for preoccupied generic names. Quaestiones Entomologicae 18: 127–259.

[B66] Bell RT Bell JR (1985) Rhysodini of the world part IV. Revisions of *Rhyzodiastes* Fairmaire and *Clinidium* Kirby, with new species in other genera (Coleoptera: Carabidae or Rhysodidae). Quaestiones Entomologicae 21: 1–172.

[B67] Benesh B (1946) A systematic revision of the Holartic genus *Platycerus* Geoffroy (Coleoptera: Lucanidae). Transactions of the American Entomological Society 72: 139–202.

[B68] Benschoter CA Cook EF (1956) A revision of the genus *Omophron* (Carabidae, Coleoptera) of North America north of Mexico. Annals of the Entomological Society of America 49: 411–429.

[B69] Bergsten J Miller KB (2006) Taxonomic revision of the Holarctic diving beetle genus *Acilius* Leach (Coleoptera: Dytiscidae). Systematic Entomology 31: 145–197. doi: 10.1111/j.1365-3113.2005.00309.x

[B70] Bieńkowski AO (2004) A review of the subgenus *Arctolina* Kontkanen, 1959 of the genus *Chrysolina* Motschulsky, 1860 (Coleoptera: Chrysomelidae: Chrysomelinae). Genus 15: 187–233.

[B71] Biström O (1997) Taxonomic revision of the genus *Hydrovatus* Motschulsky (Coleoptera, Dytiscidae). Entomologica Basiliensia 19 [1996]: 57–584.

[B72] Blackwelder RE (1941) A monograph of the genus *Trigonurus* (Coleoptera: Staphylinidae). American Museum Novitates No. 1124: 13 pp.

[B73] Blair KG (1932) The North American species of *Rhinosimus* (Col. Pythidae). The Entomologist’s Monthly Magazine 68: 253–255.

[B74] Blaisdell FE (1931) Revision of the endomychid tribe Liesthini with a description of a new genus and a new species (Coleoptera). Transactions of the American Entomological Society 56: 375–390.

[B75] Blaisdell FE (1938) A study of the species of Hispinae belonging to the genus *Stenopodius* with descriptions of new species (Coleoptera: Chrysomelidae). Transactions of the American Entomological Society 64: 421–447.

[B76] Blake DH (1927) A revision of the beetles of the genus *Oedionychis* occurring in America north of Mexico. Proceedings of the United States National Museum 70: 1–44. doi: 10.5479/si.00963801.70-2672.1

[B77] Blake DH (1933) Revision of the beetles of the genus *Disonycha* occurring in America north of Mexico. Proceedings of the United States National Museum 82: 1–66. doi: 10.5479/si.00963801.82-2969.1

[B78] Blake DH (1943) The generic position of *Hypolampsis pilosa* (Illiger) and some related new species (Coleoptera: Halticidae). Proceedings of the Entomological Society of Washington 45: 207–236.

[B79] Blake DH (1950) A revision of the beetles of the genus *Myochrous*. Proceedings of the United States National Museum 101: 1–64. doi: 10.5479/si.00963801.101-3271.1

[B80] Blake DH (1953) The chrysomelid beetles of the genus *Strabala* Chevrolat. Proceedings of the United States National Museum 103: 121–134. doi: 10.5479/si.00963801.103-3319.121

[B81] Blake DH (1955) A study of LeConte’s species of the chrysomelid genus *Graphops* with descriptions of some new species. Bulletin of the Museum of Comparative Zoology 113: 263–301.

[B82] Blake DH (1967) Revision of the beetles of the genus *Glyptoscelis* (Coleoptera: Chrysomelidae). Proceedings of the United States National Museum 123: 1–53. doi: 10.5479/si.00963801.123-3604.1

[B83] Blake DH (1970) A review of the beetles of the genus *Metachroma* Chevrolat (Coleoptera: Chrysomelidae). Smithsonian Contributions to Zoology No. 57: 111 pp.

[B84] Blake DH (1974) The costate species of *Colaspis* in the United States (Coleoptera: Chrysomelidae). Smithsonian Contributions to Zoology No. 181: 24 pp.

[B85] Böcher J (1988) The Coleoptera of Greenland. Meddelelser om Grønland 26: 1–100.

[B86] Bottimer LJ (1968) On the two species of *Bruchidius* (Coleoptera: Bruchidae) established in North America. The Canadian Entomologist 100: 139–145. doi: 10.4039/Ent100139-2

[B87] Bouchard P Bousquet Y Davies AE Alonso-Zarazaga MA Lawrence JF Lyal CHC Newton AF Reid CAM Schmitt M Ślipiński SA Smith AB (2011) Family-group names in Coleoptera (Insecta). ZooKeys 88: 1–972. doi: 10.3897/zookeys.88.807 PMC3088472 21594053

[B88] Bousquet Y (1988) *Dyschirius* of America north of Mexico: descriptions of new species with keys to species groups and species (Coleoptera: Carabidae). The Canadian Entomologist 120: 361–387. doi: 10.4039/Ent120361-4

[B89] Bousquet Y (1990) A review of the North American species of *Rhizophagus* Herbst and a revision of the Nearctic members of the subgenus *Anomophagus* Reitter (Coleoptera: Rhizophagidae). The Canadian Entomologist 122: 131–171. doi: 10.4039/Ent122131-1

[B90] Bousquet Y (Ed) (1991) Checklist of beetles of Canada and Alaska. Agriculture Canada, Ottawa, vi + 430 pp.

[B91] Bousquet Y (1996) Taxonomic revision of Nearctic, Mexican, and West Indian Oodini (Coleoptera: Carabidae). The Canadian Entomologist 128: 443–537. doi: 10.4039/Ent128443-3

[B92] Bousquet Y (1997) Description of a new species of *Clivina* Latreille from southeastern United States with a key to North American species of the *fossor* group (Coleoptera: Carabidae: Clivinini). The Coleopterists Bulletin 51: 343–349.

[B93] Bousquet Y (2002) Review of the genus *Hesperobaenus* LeConte (Coleoptera: Monotomidae) of America, north of Mexico. The Pan-Pacific Entomologist 78: 197–214.

[B94] Bousquet Y (2010) Review of the Nearctic, Mexican and West Indian (Greater Antilles) species of *Colliuris* Degeer (Coleoptera: Carabidae: Odacanthini). Zootaxa 2529: 1–39.

[B95] Bousquet Y Laplante S (2000) Taxonomic review of the Canadian species of the genus *Monotoma* Herbst (Coleoptera: Monotomidae). Proceedings of the Entomological Society of Ontario 130 [1999]: 67–96.

[B96] Bousquet Y Laplante S (2006) Coleoptera Histeridae. The Insects and Arachnids of Canada Part 24. NRC Research Press, Ottawa, xiii + 485 pp.

[B97] Bousquet Y Messer PW (2010) Redescription of *Stenolophus thoracicus* Casey (Coleoptera, Carabidae, Harpalini), a valid species. ZooKeys 53: 25–31. doi: 10.3897/zookeys.53.470 PMC3088038 21594130

[B98] Bousquet Y Skelley PE (2010) Description of a new species of *Scarites* Fabricius (Coleoptera: Carabidae) from Florida. The Coleopterists Bulletin 64: 45–49. doi: 10.1649/0010-065X-64.1.45

[B99] Bousquet Y Webster RP (2004) Review of the Nearctic species of the Holarctic subgenus *Argutor* (Coleoptera: Carabidae). The Canadian Entomologist 136: 645–660. doi: 10.4039/n04-030

[B100] Bousquet Y Webster RP (2006) Descriptions of three new species of *Bembidion* Latreille (Coleoptera: Carabidae) occurring in Canada. Zootaxa 1297: 23–35.

[B101] Bowman JR (1934) The Pselaphidae of North America. Privately published, Pittsburgh, 149 pp.

[B102] Boyle WW (1956) A revision of the Erotylidae of America north of Mexico (Coleoptera). Bulletin of the American Museum of Natural History 110: 1–172.

[B103] Brancucci M (1985) Revision du genre *Trypherus* LeConte (Coleoptera, Cantharidae). Entomologica Basiliensia 10: 251–322.

[B104] Brigham WU (1981) *Ectopria leechi*, a new false water penny from the United States (Coleoptera: Eubriidae). The Pan-Pacific Entomologist 57: 313–320.

[B105] Bright DE (1976) The Bark Beetles of Canada and Alaska. Coleoptera: Scolytidae. The Insects and Arachnids of Canada. Part 2. Agriculture Canada, Ottawa, 241 pp.

[B106] Bright DE (1981) Taxonomic monograph of the genus *Pityophthorus* Eichhoff in North and Central America (Coleoptera: Scolytidae). Memoirs of the Entomological Society of Canada No. 118: 378 pp.

[B107] Bright DE (1987) The metallic wood-boring beetles of Canada and Alaska. The Insects and Arachnids of Canada. Part 15. Agriculture Canada, Ottawa, 335 pp.

[B108] Bright DE (1993) The weevils of Canada and Alaska: Volume 1. Coleoptera: Curculionoidea, excluding Scolytidae and Curculionidae. The insects and arachnids of Canada. Part 21. Agriculture Canada, Ottawa, 217 pp.

[B109] Bright DE (1994) Revision of the genus *Sitona* (Coleoptera: Curculionidae) of North America. Annales of the Entomological Society of America 87: 277–306.

[B110] Bright DE Bouchard P (2008) Weevils of Canada and Alaska. Volume 2. Coleoptera, Curculionidae, Entiminae. NRC Research Press, Ottawa, i–xiii + 327 pp.

[B111] Brown HP (1972) Aquatic dryopoid beetles (Coleoptera) of the United States. Biota of freswater ecosystems, identification manual No. 6. United States Environmental Protection Agency, Washington D.C., 82 pp. doi: 10.5962/bhl.title.4106

[B112] Brown HP Murvosh CM (1974) A revision of the genus *Psephenus* (water-penny beetles) of the United States and Canada (Coleoptera, Dryopoidea, Psephenidae). Transactions of the American Entomological Society 100: 289–340.

[B113] Brown WJ (1942a) The American species of *Phytodecta* Kby. (Coleoptera: Chrysomelidae). The Canadian Entomologist 74: 99–105. doi: 10.4039/Ent7499-6

[B114] Brown WJ (1942b) The American species of *Entomoscelis* and *Hippuriphila* (Coleoptera, Chrysomelidae). The Canadian Entomologist 74: 172–176. doi: 10.4039/Ent74172-9

[B115] Brown WJ (1945) Food-plants and distribution of the species of *Calligrapha* in Canada, with descriptions of new species (Coleoptera, Chrysomelidae). The Canadian Entomologist 77: 117–137. doi: 10.4039/Ent77117-7

[B116] Brown WJ (1946) Notes on some species of *Canthon* and *Dichelonyx* (Coleoptera: Scarabaeidae). The Canadian Entomologist 78: 104–109. doi: 10.4039/Ent78104-5

[B117] Brown WJ (1950) The extralimital distribution of some species of Coleoptera. The Canadian Entomologist 82: 197–205. doi: 10.4039/Ent82197-10

[B118] Brown WJ (1951) The American species of *Phratora* Chev. (Coleoptera: Chrysomelidae). The Canadian Entomologist 83: 121–130. doi: 10.4039/Ent83121-5

[B119] Brown WJ (1956) The New world species of *Chrysomela* L. (Coleoptera: Chrysomelidae). The Canadian Entomologist Supplement No. 3: 54 pp.

[B120] Brown WJ (1961) Notes on North American Chrysomelidae (Coleoptera). The Canadian Entomologist 93: 967–977. doi: 10.4039/Ent93967-11

[B121] Brown WJ (1962) The American species of *Chrysolina* Mots. (Coleoptera: Chrysomelidae). The Canadian Entomologist 94: 58–74. doi: 10.4039/Ent9458-1

[B122] Brown WJ (1965) *Trachyphloeus* Germar (Coleoptera: Curculionidae) in North America. The Canadian Entomologist 97: 189–192. doi: 10.4039/Ent97189-2

[B123] Brown WJ (1966) Chrysomelinae and Curculionidae (Coleoptera); descriptions and notes. The Canadian Entomologist 98: 855–859. doi: 10.4039/Ent98855-8

[B124] Buchanan LL (1927) A short review of *Notaris* (Coleoptera: Curculionidae). Bulletin of the Brooklyn Entomological Society 22: 36–40.

[B125] Buchanan LL (1931) Synopsis of *Perigaster* (Coleoptera: Curculionidae). Journal of the Washington Academy of Sciences 21: 320–325.

[B126] Buchanan LL (1936a) The genus *Panscopus* Schoenherr (Coleoptera: Curculionidae). Smithsonian Miscellaneous Collectios No. 94: 18 pp.

[B127] Buchanan LL (1936b) Synopsis of *Lepidophorus* (Coleoptera, Curculionidae). Bulletin of the Brooklyn Entomological Society 31: 1–10.

[B128] Buchanan LL (1948) A new species of *Stenoscelis*, and notes on other Curculionidae (Coleoptera). Bulletin of the Brooklyn Entomological Society 43: 61–66.

[B129] Budarin AM (1976) Review of ground-beetles of the subgenus *Lyperopherus* Motsch. of the genus *Pterostichus* Bon. (Coleoptera, Carabidae). Trudy Zoologicheskogo Instituta Akademii Nauk SSSR 67: 32–38. [in Russian]

[B130] Burke HR (1961) Notes on *Onychylis* LeConte with descriptions of two new species (Curculionidae). The Coleopterists Bulletin 15: 1–7.

[B131] Burke HR Anderson RS (1989) Systematics of species of *Anthonomus* Germar previously assigned to *Tachypterellus* Fall and Cockerell (Coleoptera: Curculionidae). Annals of the Entomological Society of America 82: 426–437.

[B132] Butte JG (1968a) The revision of the tribe Chalepini of America North of Mexico. II. Genus *Chalepus* Thunberg (Coleoptera: Chrysomelidae). Journal of the New York Entomological Society 76: 117–133.

[B133] Butte JG (1968b) The revision of the tribe Chalepini of America North of Mexico. III. Genus *Odontota* Chevrolat (Coleoptera, Chrysomelidae). The Coleopterists Bulletin 22: 101–124.

[B134] Butte JG (1969) Revision of the tribe Chalepini of America North of Mexico. IV. Genus *Sumitrosis* Butte (Coleoptera: Chrysomelidae). Journal of the New York Entomological Society 77: 12–30.

[B135] Campbell JM (1968) A revision of the New World Micropeplidae (Coleoptera: Staphylinidae) with a rearrangement of the world species. The Canadian Entomologist 100: 225–267. doi: 10.4039/Ent100225-3

[B136] Campbell JM (1969) A revision of the New World Oxyporinae (Coleoptera: Staphylinidae). The Canadian Entomologist 101: 225–268. doi: 10.4039/Ent101225-3

[B137] Campbell JM (1973a) A revision of the genus *Tachinus* (Coleoptera: Staphylinidae) of North and Central America. Memoirs of the Entomological Society of Canada No. 90: 137 pp.

[B138] Campbell JM (1973b) New species and records of New World Micropeplinae (Coleoptera: Staphylinidae). The Canadian Entomologist 105: 569–576. doi: 10.4039/Ent105569-4

[B139] Campbell JM (1975) A revision of the genera *Coproporus* and *Cilea* (Coleoptera: Staphylinidae) of America north of Mexico. The Canadian Entomologist 107: 175–216. doi: 10.4039/Ent107175-2

[B140] Campbell JM (1976) A revision of the genus *Sepedophilus* Gistel (Coleoptera: Staphylinidae) of America north of Mexico. Memoirs of the Entomological Society of Canada No. 99: 89 pp.

[B141] Campbell JM (1978a) New species of *Oxyporus* (Coleoptera: Staphylinidae) from North America. The Canadian Entomologist 110: 805–813. doi: 10.4039/Ent110805-8

[B142] Campbell JM (1978b) A revision of the North American Omaliinae (Coleoptera: Staphylinidae). 1. The genera *Haida* Keen, *Pseudohaida* Hatch, and *Eudectoides* new genus. Memoirs of the Entomological Society of Canada No. 106: 19 pp.

[B143] Campbell JM (1978c) A revision of the North American Omaliinae (Coleoptera Staphylinidae). 2. The tribe Coryphiini. Memoirs of the Entomological Society of Canada No. 106: 20–87.

[B144] Campbell JM (1978d) *Hymenochara*, a new genus of Alleculidae (Coleoptera) based on *Mycetochara rufipes* and a new species from Arizona. The Canadian Entomologist 110: 435–441. doi: 10.4039/Ent110435-4

[B145] Campbell JM (1978e) A review of the North American species of *Mycetochara* Berthold (Coleoptera: Alleculidae). The Canadian Entomologist 110: 921–948. doi: 10.4039/Ent110921-9

[B146] Campbell JM (1979a) *Coprophilus castoris*, a new species of Staphylinidae (Coleoptera) from beaver lodges in eastern Canada. The Coleopterists Bulletin 33: 223–228.

[B147] Campbell JM (1979b) A revision of the genus *Tachyporus* Gravenhorst (Coleoptera: Staphylinidae) of North and Central America. Memoirs of the Entomological Society of Canada No. 109: 95 pp.

[B148] Campbell JM (1980) A revision of the genus *Carphacis* des Gozis (Coleoptera: Staphylinidae) of North America. The Canadian Entomologist 112: 935–953. doi: 10.4039/Ent112935-9

[B149] Campbell JM (1982a) A revision of the North American Omaliinae (Coleoptera: Staphylinidae). 3. The genus *Acidota* Stephens. The Canadian Entomologist 114: 1003–1029. doi: 10.4039/Ent1141003-11

[B150] Campbell JM (1982b) A revision of the genus *Lordithon* Thomson of North and Central America (Coleoptera: Staphylinidae). Memoirs of the Entomological Society of Canada No. 119: 114 pp.

[B151] Campbell JM (1983a) A revision of the North American Omaliinae (Coleoptera: Staphylinidae). 4. The genus *Olophrum* Erichson. The Canadian Entomologist 115: 577–622. doi: 10.4039/Ent115577-6

[B152] Campbell JM (1983b) A new species of *Pycnoglypta* Thomson (Coleoptera: Staphylinidae) from eastern Canada. The Canadian Entomologist 115: 361–370. doi: 10.4039/Ent115361-4

[B153] Campbell JM (1984a) A revision of the North American Omaliinae (Coleoptera: Staphylinidae). 5. The genera *Arpedium* Erichson and *Eucnecosum* Reitter. The Canadian Entomologist 116: 487–527. doi: 10.4039/Ent116487-4

[B154] Campbell JM (1984b) A review of the North American species of the genera *Porrhodites* Kraatz and *Orochares* Kraatz (Coleoptera: Staphylinidae). The Canadian Entomologist 116: 1227–1249. doi: 10.4039/Ent1161227-9

[B155] Campbell JM (1988) New species and records of North American *Tachinus* Gravenhorst (Coleoptera: Staphylinidae). The Canadian Entomologist 120: 231–295. doi: 10.4039/Ent120231-3

[B156] Campbell JM (1991) A revision of the genera *Mycetoporus* Mannerheim and *Ischnosoma* Stephens (Coleoptera: Staphylinidae: Tachyporinae) of North and Central America. Memoirs of the Entomological Society of Canada No. 156: 169 pp.

[B157] Campbell JM (1993) A revision of the genera *Bryoporus* Kraatz and *Bryophacis* Reitter and two new related genera from America north of Mexico (Coleoptera: Staphylinidae: Tachyporinae). Memoirs of the Entomological Society of Canada No. 166: 85 pp.

[B158] Carlson DC (1980) Taxonomic revision of *Lichnanthe* Burmeister (Coleoptera: Scarabaeidae). The Coleopterists Bulletin 34: 177–208.

[B159] Carlton CE (1983) Revision of the genus *Conoplectus* Brendel (Coleoptera: Pselaphidae). The Coleopterists Bulletin 37: 55–80.

[B160] Carlton CE (1989) Revision of the genus *Eutrichites* LeConte (Coleoptera: Pselaphidae). The Coleopterists Bulletin 43: 105–119.

[B161] Carlton CE (2003) Revision of *Reichenbachia* of eastern North America (Coleoptera, Staphylinidae, Pselaphinae). In: Leschen R Cuccudoro G (Eds) Systematics of Coleoptera: Papers celebrating the retirement of Ivan Löbl. Memoirs on Entomology, International 17: 483563.

[B162] Cartwright OL (1948) The American Species of *Pleurophorus* (Coleoptera: Scarabaeidae). Transactions of the American Entomological Society 74: 131–153.

[B163] Cartwright OL (1955) Scarab beetles of the genus *Psammodius* in the Western Hemisphere. Proceedings of the United States National Museum 104: 413–462. doi: 10.5479/si.00963801.104-3344.413

[B164] Cartwright OL (1974) *Ataenius*, *Aphotaenius*, and *Pseudataenius* of the United States and Canada (Coleoptera: Scarabaeidae: Aphodiinae). Smithsonian Contributions to Zoology No. 154: 106 pp.

[B165] Casale A (1988) Revisione degli Sphodrina (Coleoptera, Carabidae, Sphodrini). Museo Regionale di Scienze Naturali, Torino, 1024 pp.

[B166] Casari SA (1996) Systematics and phylogenetic analysis of *Alaus* Eschscholtz, 1829 (Coleoptera, Elateridae). Revista Brasileira de Entomologia 40: 249–298.

[B167] Caterino MS (1998) A phylogenetic revision of *Spilodiscus* Lewis (Coleoptera: Histeridae). Journal of Natural History 32: 1129–1168. doi: 10.1080/00222939800770571

[B168] Caterino MS Tishechkin AK (2013) A systematic revision of *Operclipygus* Marseul (Coleoptera, Histeridae, Exosternini). ZooKeys 271: 1–401. doi: 10.3897/zookeys.271.4062 PMC3652427 23717185

[B169] Cawthra EM (1957) Description of a new species of *Grypidius* Schoenherr (Col.: Curculionidae) with a key to the genus. Proceedings of the Royal Entomological Society of London, Series B, Taxonomy 26: 127–130. doi: 10.1111/j.1365-3113.1957.tb00391.x

[B170] Chandler DS (1975) A revision of *Tanarthrus* LeConte with a presentation of its mid-Cenozoic speciation (Coleoptera: Anthicidae). Transactions of the American Entomological Society 101: 319–354.

[B171] Chandler DS (1977) New *Mecynotarsus* with a key to the New World species (Coleoptera: Anthicidae). The Coleopterists Bulletin 31: 363–370.

[B172] Chandler DS (1982) A revision of North American *Notoxus* with a cladistic analysis of the New World species (Coleoptera: Anthicidae). Entomography 1: 333–438.

[B173] Chandler DS (1990) The Pselaphidae (Coleoptera) of Latimer County, Oklahoma, with revisions of four genera from eastern North America. Part 1. Faroninae and Euplectinae. Transactions of the American Entomological Society 115 [1989]: 503–529.

[B174] Chandler DS (1997) Revision of the genus *Malporus* Casey (Coleoptera: Anthicidae: Anthicinae). The Coleopterists Bulletin 51: 265–275.

[B175] Chandler DS (1999) Revision of the North American species of *Amblyderus* with a checklist of the world species (Coleoptera: Anthicidae). Transactions of the American Entomological Society 125: 269–293.

[B176] Chandler DS (2005) A revision of the New World *Cyclodinus* Mulsant & Rey (Coleoptera: Anthicidae). Transactions of the American Entomological Society 131: 1–20.

[B177] Chapin JB (1985) Revision of the genus *Mulsantina* Weise (Coleoptera: Coccinellidae). Annals of the Entomological Society of America 78: 348–368.

[B178] Chemsak JA (1963) Taxonomy and bionomics of the genus *Tetraopes* (Cerambycidae: Coleoptera). University of California Publications in Entomology No. 30: 90 pp.

[B179] Chemsak JA (1996) Illustrated revision of the Cerambycidae of North America. Volume I. Parandrinae, Spondylidinae, Aseminae, Prioninae. Wolfsgarden Books, Burbank, California, x + 150 pp.

[B180] Chemsak JA (2005) Illustrated revision of the Cerambycidae of North America. Volume II. Lepturinae. Wolfsgarden Books, Burbank, California, xv + 446 pp. + 27 pls.

[B181] Clark SM (1983) A revision of the genus *Microrhopala* (Coleoptera: Chrysomelidae) in America north of Mexico. The Great Basin Naturalist 43: 587–618.

[B182] Clark SM (1996) The genus *Scelolyperus* Crotch in North America (Coleoptera: Chrysomelidae: Galerucinae). Insecta Mundi 10: 261–280.

[B183] Clark WE (1971) A taxonomic revision of the weevil genus *Tychius* Germar in America north of Mexico (Coleoptera: Curculionidae). Brigham Young University Science Bulletin (Biological Series) 13(3): 1–39.

[B184] Clark WE (1978) The weevil genus *Sibinia* Germar: natural history, taxonomy, phylogeny, and zoogeography, with revision of the New World species (Coleoptera: Curculionidae). Quaestiones Entomologicae 14: 91–387.

[B185] Clark WE (1980) Revision of the Nearctic weevils of the genus *Lignyodes* Dejean (Coleoptera: Curculionidae). Transactions of the American Entomological Society 106: 273–326.

[B186] Clark WE (1982) Classification of the weevil tribe Lignyodini (Coleoptera, Curculionidae, Tychiinae), with revision of the genus *Plocetes*. Transactions of the American Entomological Society 108: 11–151.

[B187] Clark WE (1987a) Revision of the *Anthonomus* subgenus *Anthonomorphus* Weise (Coleoptera: Curculionidae). Quaestiones Entomologicae 23: 317–364.

[B188] Clark WE (1987b) Revision of the Nearctic species of *Pseudanthonomus* Dietz (Coleoptera: Curculionidae). The Coleopterists Bulletin 41: 263–285.

[B189] Clark WE Burke HR (2001) Revision of the weevil genus *Epimechus* Dietz (Coleoptera: Curculionidae: Anthonomini). Insecta Mundi 15: 95–116.

[B190] Clark WE Burke HR (2002) Revision of the weevil genera *Magdalinops* Dietz and *Chelonychus* Dietz (Coleoptera: Curculionidae, Anthonomini). The Coleopterists Bulletin 56: 107–122. doi: 10.1649/0010-065X(2002)056[0107:ROTWGM]2.0.CO;2

[B191] Clark WE Burke HR (2005) Revision of the subgenus *Cnemocyllus* Dietz of the weevil genus *Anthonomus* Germar (Coleoptera: Curculionidae, Anthonomini). Insecta Mundi 19: 1–54.

[B192] Cline AR Huether JP (2011) Revision of the Nearctic blister beetle genus *Tricrania* LeConte, 1860 (Coleoptera: Meloidae: Nemognathinae). Zootaxa 2832: 1–43.

[B193] Crowson RA (1966) Observation on the constitution and subfamilies of the family Melandryidae. Eos, Revista Española de Entomologia 6: 507–513.

[B194] Cuccodoro G Löbl I (1996) Revision of the rove-beetles of the genus *Megarthrus* of America north of Mexico (Coleoptera, Staphylinidae, Proteininae). Mitteilungen Münchener Entomologischen Gesellschaft 86: 145–188.

[B195] Daffner H (1988) Revision der nordamerikanischen Arten der *Cyrtusa*-Verwandtschaft (Coleoptera Leiodidae Leiodini). Annali dei Musei Civici – Rovereto 4: 269–306.

[B196] Dellacasa M Dellacasa G Gordon RD (2011) Systematic revision of the American taxa belonging to the genera *Alloblackburneus* Bordat, 2009, and *Blackburneus* Schmidt, 1913, with description of seven new species (Coleoptera: Scarabaeidae: Aphodiinae). Insecta Mundi 0204: 1–52.

[B197] Dillon LS (1956) The Nearctic components of the tribe Acanthocinini (Coleoptera: Cerambycidae). Parts I, II and III. Annals of the Entomological Society of America 49: 134–167, 207–235, 332–355.

[B198] Douglas H (2003) Revision of *Cardiophorus* (Coleoptera: Elateridae) species of eastern Canada and United States of America. The Canadian Entomologist 135: 493–548. doi: 10.4039/n02-003

[B199] Doyen JT (1973) Systematics of the genus *Coelocnemis* (Coleoptera: Tenebrionidae): a quantitative study of variation. University of California Publications in Entomology No. 73: 110 pp.

[B200] Easton AM (1955) A revision of the Nearctic species of the beetle genus *Meligethes* (Nitidulidae). Proceedings of the United States National Museum 104: 87–103. doi: 10.5479/si.00963801.104-3339.87

[B201] Edwards JG (1953) Species of the genus *Syneta* of the world (Coleoptera: Chrysomelidae). The Wasmann Journal of Biology 11: 23–82.

[B202] Eldredge KT (2012) Review of Nearctic species of *Drusilla* Leach (Coleoptera: Staphylinidae: Aleocharinae). Zootaxa 3300: 45–54.

[B203] El-Moursy AA (1958) A revision of the species of the subgenus *Plectralidus* (Harpalus, Harpalini, Carabidae) from North America. The Coleopterists Bulletin 12: 36–42.

[B204] El-Moursy AA (1970) The taxonomy of the Nearctic species of the genus *Byrrhus* Linnaeus (Coleoptera: Byrrhidae). Quaestiones Entomologicae 6: 327–338.

[B205] Endrodi S (1985) The Dynastinae of the World. Dr W. Junk, Dordrecht, 800 pp.

[B206] Endrödy-Younga S (1981) The American species of the familia Clambidae (Coleoptera, Eucinetoidea). Entomologia Generalis 7: 33–67.

[B207] Enns WR (1956) A revision of the genera *Nemognatha*, *Zonitis*, and *Pseudozonitis* (Coleoptera, Meloidae) in America north of Mexico, with a proposed new genus. The University of Kansas Science Bulletin 37: 685–909.

[B208] Erwin TL (1970a) The Nearctic species of the genus *Leistus* Frölich (Coleoptera: Carabidae). The Pan-Pacific Entomologist 46: 111–119.

[B209] Erwin TL (1970b) A reclassification of bombardier beetles and a taxonomic revision of the North and Middle American species (Carabidae: Brachinida). Quaestiones Entomologicae 6: 1–215.

[B210] Erwin TL (1975) Studies of the subtribe Tachyina (Coleoptera: Carabidae: Bembidiini), Part III: Systematics, phylogeny, and zoogeography of the genus *Tachyta* Kirby. Smithsonian Contributions to Zoology No. 208: 68 pp.

[B211] Erwin TL Kavanaugh DH (1981) Systematics and zoogeography of *Bembidion* Latreille: 1. The *carlhi* and *erasum* groups of western North America (Coleoptera: Carabidae, Bembidiini). Entomologica Scandinavica Supplementum 15: 33–72.

[B212] Evans D (1957) A revision of the genus *Poecilonota* in America north of Mexico (Coleoptera: Buprestidae). Annals of the Entomological Society of America 50: 21–37.

[B213] Fall HC (1927) A review of the North American species of *Podabrus*. Entomologica Americana 8: 65–103.

[B214] Fall HC (1931) The North American species of *Hymenorus* (Coleoptera: Alleculidae). Transactions of the American Entomological Society 57: 161–247.

[B215] Fender KM (1951) The Malthini of North America (Coleoptera: Cantharidae). American Midland Naturalist 46: 513–629. doi: 10.2307/2421804

[B216] Fender KM (1960) The Ichthyurini of North America (Coleoptera: Cantharidae). The Pan-Pacific Entomologist 36: 105–113.

[B217] Fender KM (1964) The Chauliognathini of America north of Mexico (Coleoptera: Cantharidae). Part I and Part II. Northwest Science 38: 52–64, 95–106.

[B218] Fender KM (1966) The genus *Phausis* in America north of Mexico (Coleoptera-Lampyridae). Northwest Science 40: 83–95.

[B219] Fender KM (1970) *Ellychnia* of western North America (Coleoptera: Lampyridae). Northwest Science 44: 31–43.

[B220] Fender KM (1972) Some new and little known species of Malthini from the southwestern United States (Coleoptera: Cantharidae). The Coleopterists Bulletin 26: 43–52.

[B221] Fiedler C (1939) Die Gattung *Eubulus* Kirsch (Col. Curcul. Cryptorhynch.). Deutsche Entomologische Zeitschrift 1939: 37–125.

[B222] Fisher WS (1942) A revision of the North American species of buprestid beetles belonging to the tribe Chrysobothrini. United States Department of Agriculture Miscellaneous Publications No. 470: 274 pp.

[B223] Fisher WS (1950) A revision of the North American species of beetles belonging to the family Bostrichidae. United States Department of Agriculture Miscellaneous Publications No. 698: 157 pp.

[B224] Foley IA Ivie MA (2008) A revision of the genus *Phellopsis* LeConte (Coleoptera: Zopheridae). Zootaxa 1689: 1–28.

[B225] Ford EJ (1973) A revision of the genus *Petalium* LeConte in the United States, Greater Antilles and the Bahamas (Coleoptera: Anobiidae). United States Department of Agriculture Technical Bulletin No. 1467: 40 pp.

[B226] Ford EJ (1996) The genus *Stelidota* Erichson in North America: a new species from Florida, new synonymy and lectotype designations (Coleoptera: Nitidulidae). The Coleopterists Bulletin 50: 149–153.

[B227] Foster DE (1976) Revision of the North American *Trichodes* (Herbst) (Coleoptera: Cleridae). Special Publications of the Museum Texas Tech University No. 11: 86 pp.

[B228] Frank JH (1975) A revision of the New World species of the genus *Erichsonius* Fauvel (Coleoptera: Staphylinidae). The Coleopterists Bulletin 29: 177–203.

[B229] Frank JH (1981) A revision of the New World species of the genus *Neobisnius* Ganglbauer (Coleoptera: Staphylinidae: Staphylininae). Occasional Papers of the Florida State Collection of Arthropods No. 1: 60 pp.

[B230] Frank JH (1997) Revision of the Nearctic genus *Lophioderus* Casey (Coleoptera: Scydmaenidae). The Florida Entomologist 80: 1–97. doi: 10.2307/3495983

[B231] Frank JH Habeck DH Peck SB (1987) The distribution of *Brathinus nitidus* (Coleoptera: Staphylinidae) and a key to the North American species. The Coleopterists Bulletin 41: 137–140.

[B232] Freitag R (1969) A revision of the genus *Evarthrus* LeConte (Coleoptera: Carabidae). Quaestiones Entomologicae 5: 88–211.

[B233] Génier F (1989) A revision of the genus *Hoplandria* Kraatz of America north of Mexico (Coleoptera: Staphylinidae, Aleocharinae). Memoirs of the Entomological Society of Canada Supplement No. 150: 59 pp.

[B234] Gentili E (1985) I *Laccobius* americani – I. I *Laccobius* del Canada (Coleoptera: Hydrophilidae). Osservatorio di Fisica Terrestre e Museo Antonio Stoppani del Seminario Arcivescovile di Milano (N.S.) 6: 31–45.

[B235] Gerberg EJ (1957) A revision of the New World species of powder-post beetles belonging to the family Lyctidae. United States Department of Agriculture Technical Bulletin No. 1157: 55 pp.

[B236] Gibson LP (1969) Monograph of the genus *Curculio* in the New World (Coleoptera:Curculionidae). Part I. United States and Canada. Miscellaneous Publications of the Entomological Society of America 6: 241–285.

[B237] Gidaspow T (1959) North American caterpillar hunters of the genera *Calosoma* and *Callisthenes* (Coleoptera, Carabidae). Bulletin of the American Museum of Natural History 116: 225–344.

[B238] Gidaspow T (1968) A revision of the ground beetles belonging to *Scaphinotus*, subgenus *Brennus* (Coleoptera, Carabidae). Bulletin of the American Museum of Natural History 140: 135–192.

[B239] Gidaspow T (1973) Revision of the ground beetles of American genus *Cychrus* and four subgenera of genus *Scaphinotus* (Coleoptera, Carabidae). Bulletin of the American Museum of Natural History 152: 51–102.

[B240] Gill BD Howden HF (1985) A review of the North American genus *Aphonus* LeConte (Coleoptera: Scarabaeidae: Dynastinae). The Coleopterists Bulletin 39: 119–129.

[B241] Gordon RD (1970a) A review of the genus *Microweisea* Cockerell with a description of a new genus and species of Coccinellidae from North America (Coleoptera). Proceedings of the Entomological Society of Washington 72: 207–217.

[B242] Gordon RD (1970b) A review of the genus *Delphastus* Casey (Coleoptera: Coccinellidae). Proceedings of the Entomological Society of Washington 72: 356–369.

[B243] Gordon RD (1970c) A review of the genus *Glaresis* in the United States and Canada (Coleoptera: Scarabaeidae). Transactions of the American Entomological Society 96: 499–517.

[B244] Gordon RD (1975) A revision of the Epilachninae of the Western Hemisphere (Coleoptera: Coccinellidae). US Department of Agriculture Technical Bulletin No. 1493: 409 pp.

[B245] Gordon RD (1976) The Scymnini (Coleoptera: Coccinellidae) of the United States and Canada: key to genera and revision of *Scymnus*, *Nephus* and *Diomus*. Bulletin of the Buffalo Society of Natural Sciences No. 28: 362 pp.

[B246] Gordon RD (1985) The Coccinellidae (Coleoptera) of America north of Mexico. Journal of the New York Entomological Society 93: 1–912.

[B247] Gordon RD Cartwright OL (1980) The Western Hemisphere species of *Rhyssemus* and *Trichiorhyssemus* (Coleoptera: Scarabaeidae). Smithsonian Contributions to Zoology No. 317: 29 pp.

[B248] Gordon RD Cartwright OL (1988) North American representatives of the tribe Aegialiini (Coleoptera: Scarabaeidae: Aphodiinae). Smithsonian Contributions to Zoology No. 461: 37 pp.

[B249] Gordon RD Chapin EA (1983) A revision of the New World species of *Stethorus* Weise (Coleoptera: Coccinellidae). Transactions of the American Entomological Society 109: 229–276.

[B250] Gordon RD Skelley PE (2007) A monograph of the Aphodiini inhabiting the United States and Canada (Coleoptera: Scarabaeidae: Aphodiinae). Memoirs of the American Entomological Institute 79: 1–580.

[B251] Goulet H (1983) The genera of Holarctic Elaphrini and species of *Elaphrus* Fabricius (Coleoptera: Carabidae): classification, phylogeny and zoogeography. Quaestiones Entomologicae 19: 219–482.

[B252] Goulet H Smetana A (1983) A new species of *Blethisa* Bonelli from Alaska, with proposed phylogeny, biogeography and key to known species (Coleoptera: Carabidae). The Canadian Entomologist 115: 551–558. doi: 10.4039/Ent115551-5

[B253] Green JW (1941) Taxonomic studies in *Cantharis* (Coleoptera: Cantharidae). Entomologica Americana 20 [1940]: 159–214.

[B254] Green JW (1950) The Lycidae of the United States and Canada. II. The tribe Lygistopterini (Coleoptera). Transactions of the American Entomological Society 76: 13–25.

[B255] Green JW (1951) The Lycidae of the United States and Canada. III. The tribe Platerodini (in part) (Coleoptera). Transactions of the American Entomological Society 77: 1–20.

[B256] Green JW (1952) The Lycidae of the United States and Canada. IV. The tribe Calopterini (Coleoptera). Transactions of the American Entomological Society 78: 1–19.

[B257] Green JW (1953) The Lycidae of the United States and Canada V. *Plateros* (Coleoptera). Transactions of the American Entomological Society 78: 149–181.

[B258] Green JW (1956) Revision of the Nearctic species of *Photinus* (Lampyridae: Coleoptera). Proceedings of the California Academy of Sciences 28: 561–613.

[B259] Green JW (1957) Revision of the Nearctic species of *Pyractomena* (Coleoptera: Lampyridae). The Wasmann Journal of Biology 15: 237–284.

[B260] Green JW (1961) Revision of the species of *Pyropyga* (Lampyridae). The Coleopterists Bulletin 15: 65–74.

[B261] Green JW (1966) Revision of the Nearctic species of *Silis* (Cantharidae: Coleoptera). Proceedings of the California Academy of Sciences 32: 447–513.

[B262] Grigarick AA Schuster RO (1962) Species of the genus *Batrisodes* from the Pacific slope of western North America (Coleoptera: Pselaphidae). The Pan-Pacific Entomologist 38: 199–213.

[B263] Grigarick AA Schuster RO (1967) *Reichenbachia* found in the United States west of the continental divide (Coleoptera: Pselaphidae). University of California Publications in Entomology No. 47: 45 pp.

[B264] Grigarick AA Schuster RO (1968) A revision of the genus *Cupila* Casey (Coleoptera: Pselaphidae). The Pan-Pacific Entomologist 44: 38–44.

[B265] Grigarick AA Schuster RO (1971) A revision of *Actium* Casey and *Actiastes* Casey (Coleoptera: Pselaphidae). University of California Publications in Entomology No. 67: 56 pp.

[B266] Gundersen RW (1978) Nearctic *Enochrus*. Biology, keys, descriptions and distribution (Coleoptera: Hydrophilidae). St. Cloud (MN), 54 pp.

[B267] Gusarov VI (2002) A revision of Nearctic species of the genus *Geostiba* Thomson, 1858 (Coleoptera: Staphylinidae: Aleocharinae). Zootaxa 81: 1–88. 10.11646/zootaxa.5178.3.7 36095728

[B268] Gusarov VI (2003) A revision of Nearctic species of the genera *Adota* Casey, 1910 and *Psammostiba* Yosii & Sawada, 1976 (Coleoptera: Staphylinidae: Aleocharinae). Zootaxa 185: 1–35.

[B269] Hacker HA (1968) The species of the subgenus *Leptoferonia* Casey (Coleoptera: Carabidae: *Pterostichus*). Proceedings of the United States National Museum No. 124: 61 pp.

[B270] Halstead DGH (1967) A revision of the genus *Palorus* (sens. lat.) (Coleoptera: Tenebrionidae). Bulletin of the British Museum (Natural History), Entomology 19: 59–148.

[B271] Halstead DGH (1973) A revision of the genus *Silvanus* Latreille (S.L.) (Coleoptera: Silvanidae). Bulletin of the British Museum (Natural History), Entomology 29: 39–112.

[B272] Halstead DGH (1980) A revision of the genus *Oryzaephilus* Ganglbauer, including descriptions of related genera (Coleoptera: Silvanidae). Zoological Journal of the Linnean Society 69: 271–374. doi: 10.1111/j.1096-3642.1980.tb01126.x

[B273] Hamilton RW (1971) The genus *Pselaphorhynchites* (Coleoptera: Rhynchitidae) in America north of Mexico. Annals of the Entomological Society of America 64: 982–996.

[B274] Hamilton RW (1974) The genus *Haplorhynchites* (Coleoptera: Rhynchitidae) in America north of Mexico. Annals of the Entomological Society of America 67: 787–794.

[B275] Hamilton RW (1985) The genus *Merhynchites* Sharp in America, north of Mexico. The Southwestern Entomologist 10: 49–64.

[B276] Hammond HEJ Williams DJ (2011) A revision of the genus *Callimoxys* Kraatz (Coleoptera: Cerambycidae) in America north of Mexico and review of world species. The Coleopterists Bulletin 65: 246–289. doi: 10.1649/072.065.0307

[B277] Hammond HEJ Williams DJ (2013) Casey’s conundrum, a review of the genus *Semanotus* Mulsant (Coleoptera: Cerambycidae: Cerambycinae: Callidiini) in North America. Zootaxa 3670: 101–136. doi: 10.11646/zootaxa.3670.2.1 26438930

[B278] Hammond PM (1976) A review of the genus *Anotylus* C.G. Thomson (Coleoptera: Staphylinidae). Bulletin of the British Museum (Natural History), Entomology 33: 139–187.

[B279] Hardy AR (1975) A revision of the genus *Pelidnota* of America north of Panama (Coleoptera: Scarabaeidae; Rutelinae). University of California Publications in Entomology No. 78: 43 pp.

[B280] Hardy AR (1977) A revision of the *Hoplia* of the Nearctic realm (Coleoptera: Scarabaeidae). Occasional Papers in Entomology, Sacramento No. 23: 48 pp.

[B281] Hardy AR (1978) Three new Pachydemini and a key to the species of the genus *Phobetus* (Coleoptera: Scarabaeidae). The Coleopterists Bulletin 32: 47–52.

[B282] Helava J (1978) A revision of the Nearctic species of the genus *Onthophilus* Leach (Coleoptera: Histeridae). Contributions of the American Entomological Institute 15(5): 1–43.

[B283] Helfer JR (1941) A revision of the genus *Buprestis* of North America north of Mexico (Coleoptera: Buprestidae). Entomologica Americana 21: 123–199.

[B284] Henderson LS (1940) A revision of the genus *Listronotus*: I (Curculionidae: Coleoptera). University of Kansas Science Bulletin 26: 215–337.

[B285] Herman LH (1965) Revision of *Orus*. II. Subgenera *Orus*, *Pycnorus* and *Nivorus* (Coleoptera: Staphylinidae). The Coleopterists Bulletin 19: 73–90.

[B286] Herman LH (1972a) Revision of *Bledius* and related genera. Part I. The *aequatorialis*, *mandibularis*, and *semiferrugineus* groups and two new genera (Coleoptera, Staphylinidae, Oxytelinae). Bulletin of the American Museum of Natural History 149: 113–254.

[B287] Herman LH (1972b) A revision of the rove-beetle genus *Charhyphus* (Coleoptera, Staphylinidae, Phloeocharinae). American Museum Novitates No. 2496: 16 pp.

[B288] Herman LH (1975) Revision and phylogeny of the monogeneric subfamily Pseudopsinae for the world (Staphylinidae: Coleoptera). Bulletin of the American Museum of Natural History 155: 241–318.

[B289] Herman LH (1976) Revision of *Bledius* and related genera. Part II. The *armatus*, *basalis*, and *melanocephalus* groups (Coleoptera, Staphylinidae, Oxytelinae). Bulletin of the American Museum of Natural History 157: 72–172.

[B290] Herman LH (1977) Revision and phylogeny of *Zalobius*, *Asemobius* and *Nanobius*, new genus (Coleoptera, Staphylinidae, Piestinae). Bulletin of the American Museum of Natural History 159: 45–86.

[B291] Herman LH (1983) Revision of *Bledius*. Part III. The *annularis* and *emarginatus* groups (Coleoptera, Staphylinidae, Oxytelinae). Bulletin of the American Museum of Natural History 175: 1–146.

[B292] Herman LH (1986) Revision of *Bledius*. Part IV. Classification of species groups, phylogeny, natural history, and catalogue (Coleoptera, Staphylinidae, Oxytelinae). Bulletin of the American Museum of Natural History 184: 1–367.

[B293] Hespenheide HA (1992) A review of the genus *Tachygonus* (Coleoptera: Curculionidae) north of Mexico. Proceedings of the Entomological Society of Washington 94: 1–11.

[B294] Hespenheide HA (2003) A reconsideration of *Pachyschelus schwarzi* Kerremans and a review of American *Pachyschelus* north of Mexico (Coleoptera: Buprestidae). The Coleopterists Bulletin 57: 459468. doi: 10.1649/584

[B295] Hieke F (1978) Revision der *Amara*-Untergattung *Percosia* Zimm. und Bemerkungen zu anderen *Amara*-Arten (Col., Carabidae). Deutsche Entomologische Zeitschrift 25: 215–326. doi: 10.1002/mmnd.19780250402

[B296] Hieke F (2001) Das *Amara*-Subgenus *Xenocelia* subg.n. (Coleoptera: Carabidae). Folia Heyrovskyana Supplementum No. 7: 153 pp.

[B297] Hieke F (2006) Die *Paracelia*-Gruppe der Gattung *Amara* Bonelli, 1810 (Insecta: Coleoptera: Carabidae). In: Hartmann M Weipert J (Eds) Biodiversität und Naturausstattung im Himalaya II [Biodiversity and natural heritage of the Himalaya II]. Verein der Freunde & Förderer des Naturkundemuseums Erfurt, Erfurt, 245–314. + 12 pls.

[B298] Hilsenhoff WL (1973) Notes on *Dubiraphia* (Coleoptera: Elmidae) with descriptions of five new species. Annals of the Entomological Society of America 66: 55–61.

[B299] Hinton HE (1940) A synopsis of the genus *Machronychus* Mueller (Coleoptera, Elmidae). Proceedings of the Royal Entomological Society of London, Series B, Taxonomy 9: 113–119. doi: 10.1111/j.1365-3113.1940.tb00358.x

[B300] Hinton HE (1948) A synopsis of the genus *Tribolium* Macleay, with some remarks on the evolution of its species-groups (Coleoptera, Tenebrionidae). Bulletin of Entomological Research 39: 13–56. doi: 10.1017/S0007485300024287 18865547

[B301] Hoebeke ER (1976) A revision of the genus *Xenodusa* (Staphylinidae, Aleocharinae) for North America. Sociobiology 2: 109–143.

[B302] Hoebeke ER (1985) A revision of the rove beetle tribe Falagriini of America north of Mexico (Coleoptera: Staphylinidae: Aleocharinae). Journal of the New York Entomological Society 93: 913–1018.

[B303] Hoebeke ER (1988) A new species of rove beetle, *Autalia phricotrichosa* (Coleoptera: Staphylinidae: Aleocharinae), from Mexico, with a key to the New World species of *Autalia*. The Coleopterists Bulletin 42: 87–93.

[B304] Hoebeke ER Whitehead DR (1980) New records of *Rhinoncus bruchoides* (Herbst) for the Western Hemisphere and a revised key to the North American species of the genus *Rhinoncus* (Coleoptera: Curculionidae: Ceutorhynchinae). Proceedings of the Entomological Society of Washington 82: 556–561.

[B305] Hopping R (1936) A revision of the genus *Macropogon* Motsch. The Pan-Pacific Entomologist 12: 45–48.

[B306] Howden AT (1959) A revision of the species of *Pandeleteius* Schonherr and *Pandeleteinus* Champion of America north of Mexico (Coleoptera: Curculionidae). Proceedings of the California Academy of Sciences 29: 361–421.

[B307] Howden HF (1955) Biology and taxonomy of North American beetles of the subfamily Geotrupinae, with revisions of the genera *Bolbocerosoma*, *Eucanthus*, *Geotrupes*, and *Peltotrupes* (Scarabaeidae). Proceedings of the United States National Museum 104: 151–319. doi: 10.5479/si.00963801.104-3342.151

[B308] Howden HF (1961) A revision of the New World species of *Thalycra* Erichson, with a description of a new genus and notes on generic synonymy (Coleoptera: Nitidulidae). The Canadian Entomologist Supplement No. 25: 61 pp.

[B309] Howden HF (1968) A review of the Trichiinae of North and Central America (Coleoptera: Scarabaeidae). Memoirs of the Entomological Society of Canada No. 54: 77 pp.

[B310] Howden HF Cartwright OL (1963) Scarab beetles of the genus *Onthophagus* Latreille, north of Mexico (Coleoptera: Scarabaeidae). Proceedings of the United States National Museum 114: 1–135. doi: 10.5479/si.00963801.114-3467.1

[B311] Jacques RL (1988) The potato beetles. The genus *Leptinotarsa* in North America (Coleoptera: Chrysomelidae). E.J. Brill, New York, 144 pp.

[B312] Johnson PJ (1985) A new species of *Exomella* from Idaho, with notes on the biology of *Exomella pleuralis* (Casey) (Coleoptera: Byrrhidae). The Coleopterists Bulletin 39: 151–157.

[B313] Johnson PJ (1986) A new species and a key to the Nearctic species of *Curimopsis* Ganglbauer (Coleoptera: Byrrhidae). The Coleopterists Bulletin 40: 37–43.

[B314] Johnson PJ (1991a) Taxonomic notes, new records, and a key to the adults of North American Byrrhidae (Coleoptera). Proceedings of the Entomological Society of Washington 93: 322–332.

[B315] Johnson PJ (1991b) Taxonomic reviews of *Lioon* Casey and *Listemus* Casey, with descriptions of two new species (Coleoptera: Byrrhidae). Proceedings of the Entomological Society of Washington 93: 709–718.

[B316] Johnson PJ (1997) Generic synonymy of *Arctobyrrhus* Munster and *Tylicus* Casey (Insecta: Coleoptera: Byrrhidae). Annales Zoologici, Warsaw 47: 337–340.

[B317] Johnson V Freytag PH (1982) A review of the species of *Ptilodactyla* in the United States with descriptions of three new species (Coleoptera: Ptilodactylidae). Entomological News 93: 129–135.

[B318] Jolivet P (1948) Contribution à l’étude des *Americanotimarcha* n. subg. (Col. Chrysomelidae). Bulletin du Musée Royal d’Histoire Naturelle de Belgique 24(43): 1–11.

[B319] Jolivet P (1951) Contribution à l’étude du genre *Gastrophysa* Chevrolat (Coleoptera: Chrysomelidae) (3me note) (1). Bulletin de l’Institut Royal des Sciences Naturelles de Belgique 27(21): 1–47.

[B320] Karren JB (1966) A revision of the genus *Exema* of America, north of Mexico (Chrysomelidae, Coleoptera). University of Kansas Science Bulletin 46: 647–695.

[B321] Karren JB (1972) A revision of the subfamily Chlamisinae of America north of Mexico (Coleoptera: Chrysomelidae). University of Kansas Science Bulletin 49: 877–988.

[B322] Kavanaugh DH (1986) A systematic review of amphizoid beetles (Amphizoidae: Coleoptera) and their phylogenetic relationships to other Adephaga. Proceedings of the California Academy of Sciences 44: 67–109.

[B323] Kazantsev S (2002) New cantharid genus from North America and Far East Asia (Coleoptera: Cantharidae). Elytron 15 [2001]: 43–48.

[B324] Kazantsev S (2005) A review of *Ancistronycha* Märkel with the description of *Atalantycha*, a new Nearctic genus (Coleoptera: Cantharidae). The Coleopterists Bulletin 59: 204–210. doi: 10.1649/668.1

[B325] Kingsolver JM (2004) Handbook of the Bruchidae of the United States and Canada (Insecta, Coleoptera). Volume 1 et 2. United States Department of Agriculture Technical Bulletin No. 1912.

[B326] Kingsolver JM White RE (1967) A review of the genus *Aulonium* for the United States (Coleoptera: Colydiidae). Proceedings of the Entomological Society of Washington 69: 149–154.

[B327] Kingsolver JM Whitehead DR (1976) The North and Central American species of *Meibomeus* (Coleoptera: Bruchidae: Bruchinae). United States Department of Agriculture Technical Bulletin No. 1523: 54 pp.

[B328] Kissinger DG (1970) Curculionidae tribe Ophryastini of North America (Coleoptera). Taxonomic Publications, South Lancaster (MA), 238 pp.

[B329] Klimaszewski J (1979) A revision of the Gymnusini and Deinopsini of the world. Coleoptera: Staphylinidae, Aleocharinae. Agriculture Canada, Monograph No. 25: 169 pp.

[B330] Klimaszewski J (1982) Studies of Myllaenini (Coleoptera: Staphylinidae, Aleocharinae) 1. Systematics, phylogeny, and zoogeography of Nearctic *Myllaena* Erichson. The Canadian Entomologist 114: 181–242. doi: 10.4039/Ent114181-3

[B331] Klimaszewski J (1984) A revision of the genus *Aleochara* Gravenhorst of America north of Mexico (Coleoptera: Staphylinidae, Aleocharinae). Memoirs of the Entomological Society of Canada No. 129: 211 pp.

[B332] Klimaszewski J Pelletier G (2004) Review of the *Ocalea* group of genera (Coleoptera, Staphylinidae, Aleocharinae) in Canada and Alaska: new taxa, bionomics, and distribution. The Canadian Entomologist 136: 443–500. doi: 10.4039/n03-069

[B333] Klimaszewski J Pelletier G Germain C Hebert C Humble LM Winchester NN (2001) Diversity of *Placusa* (Coleoptera: Staphylinidae, Aleocharinae) in Canada, with descriptions of two new species. The Canadian Entomologist 133: 1–47. doi: 10.4039/Ent1331-1

[B334] Klimaszewski J Pelletier G Germain C Work T Hébert C (2006) Review of *Oxypoda* species in Canada and Alaska (Coleoptera, Staphylinidae, Aleocharinae): systematics, bionomics, and distribution. The Canadian Entomologist 138: 737–852. doi: 10.4039/n05-064

[B335] Klimaszewski J Pelletier G Majka C (2004) A revision of Canadian *Leptusa* Kraatz (Col., Staphylinidae, Aleocharinae): new species, new distribution records, key and taxonomic considerations. Belgian Journal of Entomology 6: 3–42.

[B336] Klimaszewski J Pohl G Pelletier G (2003) Revision of the Nearctic *Silusa* (Coleoptera, Staphylinidae, Aleocharinae). The Canadian Entomologist 135: 159–186. doi: 10.4039/n02-027

[B337] Klimaszewski J Savard K Pelletier G Webster R (2008a) Species review of the genus *Gnypeta* Thomson from Canada, Alaska and Greenland (Coleoptera, Staphylinidae, Aleocharinae): systematics, bionomics and distribution. ZooKeys (Special Issue) 2: 11–84.

[B338] Klimaszewski J Webster R Assing V Savard K (2008b) *Diglotta mersa* (Haliday) and *Halobrecta flavipes* Thomson, two new species for the Canadian fauna (Coleoptera, Staphylinidae, Aleocharinae). ZooKeys (Special Issue) 2: 175–188.

[B339] Klimaszewski J Webster RP Savard K (2009a) First record of the genus *Schistoglossa* Kraatz from Canada with descriptions of seven new species (Coleoptera, Staphylinidae, Aleocharinae). ZooKeys 22: 45–79. doi: 10.3897/zookeys.22.153

[B340] Klimaszewski J Webster RP Savard K Couture J (2009b) First record of the genus *Alisalia* Casey from Canada, description of two new species, and a key to all Nearctic species of the genus (Coleoptera, Staphylinidae, Aleocharinae). ZooKeys 25: 1–18. doi: 10.3897/zookeys.25.280

[B341] Kuschel G (1952) Revision de *Lissorhoptrus* LeConte y géneros vecinos de América (Ap. 11 de Coleoptera Curculionidae). Revista Chilena de Entomologia 1: 23–74.

[B342] Kuschel G (1989) The Nearctic Nemonychidae (Coleoptera: Curculionoidea). Entomologica Scandanavica 20: 121–171. doi: 10.1163/187631289X00276

[B343] La Rivers I (1949) A new species of *Microcylloepus* from Nevada (Coleoptera: Dryopidae). Entomological News 60: 205–209.

[B344] Larson DJ (1969) A revision of the genera *Philophuga* Motschulsky and *Tecnophilus* Chaudoir with notes on the North American Callidina (Coleoptera: Carabidae). Quaestiones Entomologicae 5: 15–84.

[B345] Larson DJ (1987) Revision of North American species of *Ilybius* Erichson (Coleoptera: Dytiscidae), with systematic notes on Palaearctic species. Journal of the New York Entomological Society 95: 341–413.

[B346] Larson DJ Alarie Y Roughley RE (2000) Predaceous diving beetles (Coleoptera: Dytiscidae) of the Nearctic Region, with emphasis on the fauna of Canada and Alaska. NRC Research Press, Ottawa, xiv + 982 pp.

[B347] Larson DJ Roughley RE (1990) A review of the species of *Liodessus* Guignot of North America north of Mexico with the description of a new species (Coleoptera: Dytiscidae). Journal of the New York Entomological Society 98: 233–245.

[B348] Lawrence JF (1967) Delimitation of the genus *Ceracis* (Coleoptera: Ciidae) with a revision of North American species. Bulletin of the Museum of Comparative Zoology 136: 91–144.

[B349] Lawrence JF (1971) Revision of the North American Ciidae (Coleoptera). Bulletin of the Museum of Comparative Zoology 142: 419–522.

[B350] Lawrence JF Hlavac TF (1979) Review of the Derodontidae (Coleoptera: Polyphaga) with new species from North America and Chile. The Coleopterists Bulletin 33: 369–414.

[B351] Lawrence JF Stephan K (1975) The North American Cerylonidae (Coleoptera: Clavicornia). Psyche 82: 131–166. doi: 10.1155/1975/16703

[B352] Ledoux G Roux P (2005) *Nebria* (Coleoptera, Nebriidae). Faune mondiale. Muséum [&] Société Linnaeusenne de Lyon, Lyon, 976 pp.

[B353] Lepesme P (1949) Revision des *Dermestes* (Col. Dermestidae). Annales de la Société Entomologique de France 115: 37–68.

[B354] LeSage L (1986) A taxonomic monograph of the Nearctic galerucine genus *Ophraella* Wilcox (Coleoptera: Chrysomelidae). Memoirs of the Entomological Society of Canada No. 133: 75 pp.

[B355] LeSage L Paquin P (1996) Identification keys for *Aphthona* flea beetles (Coleoptera: Chrysomelidae) introduced in Canada for the control of spurge (*Euphorbia* spp., Euphorbiaceae). The Canadian Entomologist 128: 593–603. doi: 10.4039/Ent128593-4

[B356] Lewis AE (1986) The *Sternidius* of America north of Mexico (Coleoptera: Cerambycidae). The Pan-Pacific Entomologist 62: 171–202.

[B357] Liebherr JK (1991) Phylogeny and revision of the *Anchomenus* clade: the genera *Tetraleucus*, *Anchomenus*, *Sericoda*, and *Elliptoleus* (Coleoptera: Carabidae: Platynini). Bulletin of the American Museum of Natural History No. 202: 163 pp.

[B358] Liebherr JK (1994) Identification of New World *Agonum*, review of the Mexican fauna, and description of *Incagonum*, new genus, from South America (Coleoptera: Carabidae: Platynini). Journal of the New York Entomological Society 102: 1–55.

[B359] Liebherr JK Will KW (1996) New North American *Platynus* Bonelli (Coleoptera: Carabidae), a key to species north of Mexico, and notes on species from the southwestern United States. The Coleopterists Bulletin 50: 301–320.

[B360] Liljeblad E (1945) Monograph of the family Mordellidae (Coleoptera) of North America, north of Mexico. Miscellaneous Publications, University of Michigan, Museum of Zoology No. 62: 229 pp.

[B361] Lindroth CH (1956) A revision of the genus *Synuchus* Gyllenhal (Coleoptera: Carabidae) in the widest sense, with notes on *Pristosia* Motschulsky (*Eucalathus* Bates) and *Calathus* Bonelli. The Transactions of the Royal Entomological Society of London 108: 485–585. doi: 10.1111/j.1365-2311.1956.tb01274.x

[B362] Lindroth CH (1961–1969) The ground–beetles (Carabidae, excl. Cicindelinae) of Canada and Alaska. Opuscula Entomologica Supplementum 20 (1961): 1–200; 24 (1963): 201–408; 29 (1966): 409–648; 33 (1968): 649–944; 34 (1969a): 945–1192; 35 (1969b): iii–xlviii.

[B363] Linsley EG (1962a) The Cerambycidae of North America, part II. Taxonomy and classification of the Parandrinae, Prioninae, Spondylinae, and Aseminae. University of California Publications in Entomology No. 19: 102 pp.

[B364] Linsley EG (1962b) The Cerambycidae of North America, part III. Taxonomy and classification of the subfamily Cerambycinae, tribes Opsimini through Megaderini. University of California Publications in Entomology No. 20: 188 pp.

[B365] Linsley EG (1963) The Cerambycidae of North America, part IV. Taxonomy and classification of the subfamily Cerambycinae, tribes Elaphidionini through Rhinotragini. University of California Publications in Entomology No. 21: 165 pp.

[B366] Linsley EG (1964) The Cerambycidae of North America, part V. Taxonomy and classification of the subfamily Cerambycinae, tribes Callichromini through Ancylocerini. University of California Publications in Entomology No. 22: 197 pp.

[B367] Linsley EG Chemsak JA (1972) The Cerambycidae of North America, part VI, no. 1. Taxonomy and classification of the subfamily Lepturinae. University of California Publications in Entomology No. 69: 138 pp.

[B368] Linsley EG Chemsak JA (1976) The Cerambycidae of North America, part VI, no. 2. Taxonomy and classification of the subfamily Lepturinae. University of California Publications in Entomology No. 80: 186 pp.

[B369] Linsley EG Chemsak JA (1985) The Cerambycidae of North America, part VII, no. 1: Taxonomy and classification of the subfamily Lamiinae, tribes Parmenini through Acanthoderini. University of California Publications in Entomology No. 102: 258 pp.

[B370] Linsley EG Chemsak JA (1995) The Cerambycidae of North America, part VII, no. 2: Taxonomy and classification of the subfamily Lamiinae, tribes Acanthocinini through Hemilophini. University of California Publications in Entomology No. 114: 292 pp.

[B371] Löbl I Stephan K (1993) A review of the species of *Baeocera* Erichson (Coleoptera, Staphylinidae, Scaphidiinae) of America north of Mexico. Revue Suisse de Zoologie 100: 675–733.

[B372] Lohse GA Klimaszewski J Smetana A (1990) Revision of arctic Aleocharinae of North America (Coleoptera: Staphylinidae). The Coleopterists Bulletin 44: 121–202.

[B373] Luginbill P Painter HR (1953) May beetles of the United States and Canada. Department of Agriculture Technical Bulletin No. 1060: 102 pp.

[B374] MacRae TC (1991) The Buprestidae (Coleoptera) of Missouri. Insecta Mundi 5: 101126.

[B375] Maddison DR (1993) Systematics of the Holarctic beetle subgenus *Bracteon* and related *Bembidion* (Coleoptera: Carabidae). Bulletin of the Museum of Comparative Zoology 153: 143–299.

[B376] Maddison DR (2008) Systematics of the North American beetle subgenus *Pseudoperyphus* (Coleoptera: Carabidae: Bembidion) based upon morphological, chromosomal, and molecular data. Annals of Carnegie Museum 77: 147–193. doi: 10.2992/0097-4463-77.1.147

[B377] Madge RB (1967) A revision of the genus *Lebia* Latreille in America north of Mexico (Coleoptera, Carabidae). Quaestiones Entomologicae 3: 139–242.

[B378] Majka CG Anderson RS (2007) The genus *Tropiphorus* (Coleoptera: Curculionidae: Entiminae) in North America. The Coleopterists Bulletin 61: 487–489. doi: 10.1649/0010-065X(2007)61[487:TGTCCE]2.0.CO;2

[B379] Mank EW (1938) A revision of the genus *Zilora*. Psyche 45: 101–104. doi: 10.1155/1938/87695

[B380] Mank EW (1939a) A review of the genus *Serropalpus*, (Coleoptera, Melandryidae). The Canadian Entomologist 71: 237–239. doi: 10.4039/Ent71237-11

[B381] Mank EW (1939b) *Scotochroa* and a closely allied new genus *Scotochroides*, (Coleoptera, Melandryidae). The Canadian Entomologist 71: 181–183. doi: 10.4039/Ent71181-8

[B382] Marek PE Kavanaugh DH (2005) The evolutionary relationships of North American *Diplous* Motschulsky (Coleoptera: Carabidae: Patrobini) inferred from morphological and molecular evidence. Invertebrate Systematics 19: 145–168. doi: 10.1071/IS04011

[B383] Marsh GA Schuster RO (1962) A revision of the genus *Sonoma* Casey (Coleoptera: Pselaphidae). The Coleopterists Bulletin 16: 33–56.

[B384] Marshall JD (1970) *Isomira* Mulsant in America north of Mexico (Coleoptera: Alleculidae): species of eastern North America concluded. The Coleopterists Bulletin 24: 88–95.

[B385] Martins UR (1970) Monografia da tribo Ibidionini (Coleoptera, Cerambycinae). Arquivos de Zoologia, Sao Paulo 16: 1205–1342.

[B386] Marx EJF (1957) A review of the subgenus *Donacia* in the Western Hemisphere (Coleoptera, Donaciidae). Bulletin of the American Museum of Natural History 112: 195–278.

[B387] Matta JF Wolfe GW (1981) A revision of the subgenus *Heterosternuta* Strand of *Hydroporus* Clairville (Coleoptera: Dytiscidae). The Pan-Pacific Entomologist 57: 176–219.

[B388] Matthews EG (1961) A revision of the genus *Copris* Muller of the Western Hemisphere (Coleoptera: Scarabaeidae). Entomologica Americana 41: 1–139.

[B389] McHugh JV (1993) A revision of *Eurysphindus* LeConte (Coleoptera: Cucujoidea: Sphindidae) and a review of sphindid classification and phylogeny. Systematic Entomology 18: 57–92. doi: 10.1111/j.1365-3113.1993.tb00654.x

[B390] McKey-Fender D (1950) Notes on Cantharis III. The Pan-Pacific Entomologist 26: 25–33; 61–79.

[B391] Mignot EC (1971) Review of *Blepharida* Chevrolat (Chrysomelidae: Alticinae) in America north of Mexico. The Coleopterists Bulletin 25: 9–16.

[B392] Miller DC (1974) Revision of the New World *Chaetarthria* (Coleoptera: Hydrophilidae). Entomologica Americana 49: 1–123.

[B393] Miller KB (1998) Revision of the Nearctic *Liodessus affinis* (Say 1823) species group (Coleoptera: Dytiscidae, Hydroporinae, Bidessini). Entomologica Scandinavica 29: 281–314. doi: 10.1163/187631298X00113

[B394] Miller KB (2001) Revision and phylogenetic analysis of the New World genus *Neoclypeodytes* Young (Coleoptera: Dytiscidae: Hydroporinae: Bidessini). Systematic Entomology 26: 87–123. doi: 10.1046/j.1365-3113.2001.00144.x

[B395] Miller KB Wheeler QD (2005a) Two new genera of Agathidiini from the Nearctic and Neotropical Regions (Coleoptera: Leiodidae). The Coleopterists Bulletin 58 [2004]: 466–487.

[B396] Miller KB Wheeler QD (2005b) Slime-mold beetles of the genus *Agathidium* Panzer in North and Central America, Part II. Coleoptera: Leiodidae. Bulletin of the American Museum of Natural History No. 291: 167 pp.

[B397] Miller WV (1988) Two new species of *Heterocerus* from North America, with notes on related species (Coleoptera: Heteroceridae). The Coleopterists Bulletin 42: 313–320.

[B398] Millers I Benjamin DM Warner RE (1963) A new *Hylobius* weevil associated with jack pine stand deterioration (Coleoptera: Curculionidae). The Canadian Entomologist 95: 18–22. doi: 10.4039/Ent9518-1

[B399] Moldenke AR (1970) A revision of the Clytrinae of North America north of the Isthmus of Panama (Coleoptera: Chrysomelidae). Stanford University, Stanford (CA), 310 pp.

[B400] Moore IM (1975) The distribution of *Siagonium* (Coleoptera: Staphylinidae) in North America. Journal of the Kansas Entomological Society 48: 96–100.

[B401] Moore IM Legner EF (1975) Revision of the genus *Endeodes* LeConte with a tabular key to the species (Coleoptera: Melyridae). Journal of the New York Entomological Society 83: 70–81.

[B402] Mousseau T Roughley RE (2007) Taxonomy, classification, reconstructed phylogeny and biogeography of Nearctic species of *Brychius* Thomson (Coleoptera: Haliplidae). The Coleopterists Bulletin 61: 351–397. doi: 10.1649/0010-065X(2007)61[351:TCRPAB]2.0.CO;2

[B403] Munroe DD Smith RF (1980) A revision of the systematics of *Acalymna* *sensu stricto* Barber (Coleoptera: Chrysomelidae) from North America including Mexico. Memoirs of the Entomological Society of Canada No. 112: 92 pp.

[B404] Muona J (2000) A revision of the Nearctic Eucnemidae. Acta Zoologica Fennica 212: 1–106.

[B405] Musgrave PN (1935) A synopsis of the genus *Helichus* Erichson in the United States and Canada, with description of a new species (Coleoptera: Dryopidae). Proceedings of the Entomological Society of Washington 37: 137–145.

[B406] Neboiss A (1984) Reclassification of *Cupes* Fabricius (s. lat.), with descriptions of new genera and species (Cupedidae: Coleoptera). Systematic Entomology 9: 443–477. doi: 10.1111/j.1365-3113.1984.tb00520.x

[B407] Nelson GH (1975) A revision of the genus *Dicerca* in North America (Coleoptera:Buprestidae). Entomologische Arbeiten aus dem Museum G. Frey, Tutzing bei München 26: 87–180.

[B408] Nelson GH (1978) A review of the genus *Ptosima* in North America (Coleoptera: Buprestidae). The Coleopterists Bulletin 32: 327336.

[B409] Nelson GH (1979) A new species of *Actenodes* from the United States with a key to the species (Coleoptera: Buprestidae). The Coleopterists Bulletin 33: 8791.

[B410] Nicolay AS Weiss HB (1923) The group Traches in North America, part II. The genus *Brachys*. Journal of the New York Entomological Society 31: 5976.

[B411] Nikitsky NB (2004) The beetles of the subfamily Tetratominae Billberg, 1820 (Coleoptera, Tetratomidae) of the world fauna. Byulleten’ Moskovskogo Obshchestva Ispytatelei Prirody Otdel Biologicheskii 109: 25–36. [in Russian]

[B412] Nikitsky NB (2007) The beetles of the genus *Holostrophus* Horn, 1888 (Coleoptera, Tetratomidae, Eustrophinae, Holostrophini) of the world fauna [in Russian]. Byulleten’ Moskovskogo Obshchestva Ispytatelei Prirody Otdel Biologicheskii 112: 13–30.

[B413] Nikitsky NB Belov VV (1982) The false darkling beetle genus *Lederia* Rtt. (Coleoptera, Melandryidae). Folia Entomologica Hungarica 43: 111–123.

[B414] Noonan GR (1968) A revision of the genus *Dicheirus* Mannerheim 1843 (Col. Carabidae). Opuscula Entomologica 33: 281–304.

[B415] Noonan GR (1973) The anisodactylines (Insecta: Coleoptera: Carabidae: Harpalini): classification, evolution, and zoogeography. Quaestiones Entomologicae 9: 266–480.

[B416] Noonan GR (1991) Classification, cladistics, and natural history of native North American *Harpalus* Latreille (Insecta: Coleoptera: Carabidae: Harpalini), excluding subgenera *Glanodes* and *Pseudophonus*. Thomas Say Foundation Monographs No. 13: viii + 310 pp.

[B417] Noonan GR (1996) Classification, cladistics, and natural history of species of the subgenus *Anisodactylus* Dejean (Insecta: Coleoptera: Carabidae: Harpalini: Anisodactylus). Milwaukee Public Museum Contributions in Biology and Geology No. 89: iii + 210 pp.

[B418] Noonan GR (2001) Systematics and cladistics of the North American subgenus *Anadaptus* Casey (genus *Anisodactylus* Dejean) and a geographic information system analysis of the biogeography of included species. Annals of the Entomological Society of America 94: 301–332. doi: 10.1603/0013-8746(2001)094[0301:SACOTN]2.0.CO;2

[B419] O’Brien CW (1970a) A taxonomic revision of the genus *Gerstaeckeria* north of Mexico (Coleoptera: Curculionidae). Annals of the Entomological Society of America 63: 255–272.

[B420] O’Brien CW (1970b) A taxonomic revision of the weevil genus *Dorytomus* in North America (Coleoptera: Curculionidae). University of California Publications in Entomology No. 60: 80 pp.

[B421] O’Brien CW (1981) The larger (4.5 + mm.) *Listronotus* of America, north of Mexico (Cylindrorhininae, Curculionidae, Coleoptera). Transactions of the American Entomological Society 107: 69–123.

[B422] O’Brien CW Ciegler JC Girón JC (2010) Weevils of the genus *Cercopeus* Schoenherr from South Carolina, USA (Coleoptera: Curculionidae: Entiminae). Insecta Mundi 0141: 1–29.

[B423] Opitz W (2011) Classification, natural history, and evolution of Epiphloeinae (Coleoptera, Cleridae). Part X. The genus *Madoniella* Pic, 1935. Entomologica Basiliensia et Collectionis Frey 33: 133–248.

[B424] Opitz W (2013) Revision of the New World genus *Neorthopleura* Barr (Coleoptera: Cleridae). Annales de la Société Entomologique de France (N.S.) 49: 1–35.

[B425] Orth RE Moore I (1980) A revision of the species of *Cafius* Curtis from the west coast of North America with notes of the east coast species (Coleoptera: Staphylinidae). Transactions of the San Diego Society of Natural History 19: 181–211.

[B426] Oygur S Wolfe GW (1991) Classification, distribution, and phylogeny of North American (north of Mexico) species of *Gyrinus* Müller (Coleoptera: Gyrinidae). Bulletin of the American Museum of Natural History 207: 1–97.

[B427] Pace R (1989) Monografia del genere *Leptusa* Kraatz (Coleoptera, Staphylinidae). Memorie del Museo Civico di Storia Naturale di Verona (2) (A: Biologie) 8: 1–307.

[B428] Pacheco F (1964) Sistematica, filogenia y distribucion de los Heteroceridos de America (Coleoptera: Heteroceridae). Monografias del Colegio de Post–Graduados No. 1: 155 pp.

[B429] Papp CS (1962) An illustrated and descriptive catalogue of the Ptinidae of North America. Deutsche Entomologische Zeitschrift 9: 367–423. doi: 10.1002/mmnd.19620090502

[B430] Park O (1947) Observations on *Batrisodes* (Coleoptera: Pselaphidae), with particular reference to the American species east of the Rocky Mountains. Bulletin of the Chicago Academy of Sciences 8: 45–132.

[B431] Park O (1949) New species of Nearctic pselaphid beetles and a revision of the genus *Cedius*. Bulletin of the Chicago Academy of Sciences 8: 315–343.

[B432] Park O (1956) New or little known species of pselaphid beetles from southeastern United States. Journal of the Tennessee Academy of Science 31: 54–100.

[B433] Park O (1958) New or little known species of pselaphid beetles chiefly from southeastern United States. Journal of the Tennessee Academy of Science 33: 39–74.

[B434] Park O Wagner JA (1962) Family Pselaphidae. In: Hatch MH (Ed) The beetles of the Pacific Northwest. Part III: Pselaphidae and Diversicornia I. University of Washington Press, Seattle, 4–31.

[B435] Parry RH (1974) Revision of the genus *Dibolia* Latreille in America North of Mexico (Coleoptera: Chrysomelidae). Canadian Journal of Zoology 52: 1317–1354. doi: 10.1139/z74-172

[B436] Parry RH (1986) The systematics and biology of the flea beetle genus *Crepidodera* (Coleoptera: Chrysomelidae) in America north of Mexico. Insecta Mundi 1: 156–196.

[B437] Parry RH Howden HF (1975) A new species of *Colopterus* from Florida (Coleoptera, Nitidulidae). The Coleopterists Bulletin 29: 275–279.

[B438] Parsons CT (1943) A revision of Nearctic Nitidulidae (Coleoptera). Bulletin of the Museum of Comparative Zoology 92: 121–278.

[B439] Parsons CT (1975a) A key to Nearctic *Statira* and *Arthromacra* (Lagriidae). The Coleopterists Bulletin 29: 211–226.

[B440] Parsons CT (1975b) Revision of Nearctic Mycetophagidae (Coleoptera). The Coleopterists Bulletin 29: 93–108.

[B441] Paśnik G (2006) A revision of the World species of the genus *Tachyusa* Erichson, 1837 (Coleoptera, Staphylinidae: Aleocharinae). Zootaxa 1146: 1–152.

[B442] Pearson DL Knisley CB Kazilek CJ (2006) A field guide to the tiger beetles of the United States and Canada: identification, natural history, and distribution of the Cicindelidae. Oxford University Press, New York, vi + 227 pp. + 24 pls.

[B443] Peck SB (1973) A systematic revision and the evolutionary biology of the *Ptomaphagus (Adelops)* beetles of North America (Coleoptera; Leiodidae; Catopinae) with emphasis on cave-inhabiting species. Bulletin of the Museum of Comparative Zoology 145: 29–162.

[B444] Peck SB (1982) A review of the ectoparasitic *Leptinus* beetles of North America (Coleoptera: Leptinidae). Canadian Journal of Zoology 60: 1517–1527. doi: 10.1139/z82-201

[B445] Peck SB (1998) Revision of the *Colenis* of America north of Mexico (Coleoptera: Leiodidae: Leiodinae: Pseudoliodini). The Canadian Entomologist 130: 55–65. doi: 10.4039/Ent13055-1

[B446] Peck SB Cook J (2002) Systematics, distributions, and bionomics of the small carrion beetles (Coleoptera: Leiodidae: Cholevinae: Cholevini) of North America. The Canadian Entomologist 134: 723–787. doi: 10.4039/Ent134723-6

[B447] Peck SB Cook J (2007) Systematics, distributions, and bionomics of the *Neoeocatops* gen. nov. and *Nemadus* of North America (Coleoptera: Leiodidae: Cholevinae: Anemadini). The Canadian Entomologist 139: 87–117. doi: 10.4039/n05-091

[B448] Peck SB Cook J (2009) Review of the Sogdini of North and Central America (Coleoptera: Leiodidae: Leiodinae) with descriptions of fourteen new species and three new genera. Zootaxa 2102: 1–74.

[B449] Peck SB Cook J (2011) Systematics, distributions and bionomics of the Catopocerini (eyeless soil fungivore beetles) of North America (Coleoptera: Leiodidae: Catopocerinae). Zootaxa 3077: 1–118.

[B450] Peck SB Cook J (2013) A revision of the species of *Anogdus* LeConte of the United States and Canada (Coleoptera: Leiodidae: Leiodinae: Leiodini). Insecta Mundi 0290: 1–27.

[B451] Peck SB Stephan K (1996) A revision of the genus *Colon* Herbst (Coleoptera; Leiodidae; Coloninae) of North America. The Canadian Entomologist 128: 667–741. doi: 10.4039/Ent128667-4

[B452] Perkins PD (1980) Aquatic beetles of the family Hydraenidae in the Western Hemisphere: classification, biogeography and inferred phylogeny (Insecta: Coleoptera). Quaestiones Entomologicae 16: 3–554.

[B453] Pierce WD (1975) The sand dune weevils of the genus *Trigonoscuta* with a correlation of their anatomy to the geological history of our coast lines. John G. Shanafelt, Orange (CA), 162 pp.

[B454] Pinto JD (1980) Behavior and taxonomy of the *Epicauta maculata* group (Coleoptera, Meloidae). University of California Publications in Entomology No. 89: 111 pp.

[B455] Pinto JD (1991) The taxonomy of North American *Epicauta* (Coleoptera: Meloidae) with a revision of the nominate subgenus and a survey of courtship behavior. University of California Publications in Entomology No. 110: 372 pp.

[B456] Pinto JD (2009) A taxonomic review of the genus *Gnathium* Kirby (Coleoptera: Meloidae). Transactions of the American Entomological Society 135: 1–58. doi: 10.3157/061.135.0201

[B457] Pinto JD Selander RB (1970) The bionomics of blister beetles of the genus *Meloe* and a classification of the New World species. Illinois Biological Monographs No. 42: 222 pp.

[B458] Pollock DA (1991) Natural history, classification, reconstructed phylogeny and geographic history of *Pytho* Latreille (Coleoptera: Heteromera: Pythidae). Memoirs of the Entomological Society of Canada No. 154: 104 pp.

[B459] Pollock DA (1993) A new species of *Mycterus* Clairville (Coleoptera: Mycteridae) from Florida, with a key to species of America north of Mexico. The Coleopterists Bulletin 47: 309–314.

[B460] Pollock DA (2008) Review of the Canadian Eustrophinae (Coleoptera, Tetratomidae). Zookeys (Special Issue) 2: 261–290. doi: 10.3897/zookeys.2.30 PMC3345898 22611332

[B461] Pollock DA Majka CG (2012) Review of the Nearctic genus *Lacconotus* LeConte (Coleoptera, Mycteridae, Eurypinae). ZooKeys 162: 1–24. doi: 10.3897/zookeys.162.1998 PMC3253660 22303123

[B462] Potts RW (1977) Revision of the Scarabaeidae: Anomalinae. 3. A key to the species of *Anomala* of America north of Mexico. The Pan-Pacific Entomologist 53: 129–134.

[B463] Prena J (2008) Review of *Odontocorynus* Schönherr (Coleoptera: Curculionidae: Baridinae) with descriptions of four new species. The Coleopterists Bulletin 62: 243–277. doi: 10.1649/1074.1

[B464] Puthz V (1974a) A new revision of the Nearctic *Edaphus*-species and remarks on other North American Euaesthetinae (Coleoptera: Staphylinidae). Revue Suisse de Zoologie 81: 911–932.

[B465] Puthz V (1974b) Die zweite Art der Gattung *Stictocranius* LeConte: *Stictocranius chinensis* nov. spec. (Coleoptera: Staphylinidae). 10. Beitrag zur Kenntnis der Euaesthetinen. Entomologische Blätter für Biologie und Systematik der Käfer 70: 35–38.

[B466] Quate LW Thompson SE (1967) Revision of click beetles of genus *Melanotus* in America north of Mexico (Coleoptera: Elateridae). Proceedings of the United States National Museum 121(3568): 1–83. doi: 10.5479/si.00963801.121-3568.1

[B467] Rabaglia RJ Dole SA Cognato AI (2006) Review of American Xyleborina (Coleoptera: Curculionidae: Scolytinae) occurring North of Mexico, with an illustrated key. Annals of the Entomological Society of America 99: 1034–1056. doi: 10.1603/0013-8746(2006)99[1034:ROAXCC]2.0.CO;2

[B468] Reichardt H (1967) A monographic revision of the American Galeritini (Coleoptera, Carabidae). Arquivos de Zoologia 15: 1–176.

[B469] Reichardt H (1976) Monograph of the New World Nosodendridae and notes on the Old World forms (Coleoptera). Papeis Avulsos do Departamento de Zoologia 29: 185–220.

[B470] Riley EG (1985a) Identification of *Cassida atripes* LeConte 1859 and *Coptocycla bisignata* Boheman 1855, two American tortoise beetles. Journal of the Kansas Entomological Society 58: 53–61.

[B471] Riley EG (1985b) Review of the North American species of *Glyphuroplata* Uhmann, 1940 (Coleoptera: Chrysomelidae: Hispinae). Journal of the Kansas Entomological Society 58: 428–436.

[B472] Riley EG (1986a) Review of the tortoise beetle genera of the tribe Cassidini occurring in America north of Mexico (Coleoptera, Chrysomelidae, Cassidinae). Journal of the New York Entomological Society 94: 98–114.

[B473] Riley EG (1986b) Notes on *Cassida relicta*, a tortoise beetle endemic to North America, with a key to Nearctic species of *Cassida* (Coleoptera: Chrysomelidae). Entomological News 97: 141–146.

[B474] Rivnay E (1929) Revision of the Rhipiphoridae of North and Central America (Coleoptera). Memoirs of the American Entomological Society No. 6: 68 pp.

[B475] Roache LC (1961) A revision of the North American elaterid beetles of the tribe Elaterini (Coleoptera: Elateridae). Transactions of the American Entomological Society 86: 275–324.

[B476] Roache LC (1963) A revision of the genus *Oxygonus* LeConte with a description of one new species (Coleoptera: Elateridae) (Tribe: Agriotini). The Coleopterists Bulletin 17: 101–108.

[B477] Robinson M (1948) A review of the species of *Canthon* inhabiting the United States (Scarabaeidae: Coleoptera). Transactions of the American Entomological Society 74: 83–99.

[B478] Roughley RE (1990) A systematic revision of species of *Dytiscus* Linnaeus (Coleoptera: Dytiscidae). Part 1. Classification based on adult stage. Quaestiones Entomologicae 26: 383–557.

[B479] Roughley RE Pengelly DH (1981) Classification, phylogeny and zoogeography of *Hydaticus* Leach (Coleoptera: Dytiscidae). Quaestiones Entomologicae 17: 249–314.

[B480] Sanderson MW (1948) Larval, pupal, and adult stages of North American *Physonota* (Chrysomelidae). Annals of the Entomological Society of America 41: 468–477.

[B481] Saylor LW (1945) Revision of the scarab beetles of the genus *Dichelonyx*. Bulletin of the Brooklyn Entomological Society 40: 137–158.

[B482] Scherer G (1974) Review of North American species of *Orthaltica* with new generic synonymy (Coleoptera: Chrysomelidae: Alticinae). The Coleopterists Bulletin 28: 65–72.

[B483] Schoof HF (1942) The genus *Conotrachelus* Dejean (Coleoptera, Curculionidae) in the north central United States. Illinois Biological Monographs No. 19: 170 pp.

[B484] Schultz WT (1976) Review of the genus *Spintherophyta* (Coleoptera: Chrysomelidae) in North America north of Mexico. Annals of the Entomological Society of America 69: 877–881.

[B485] Schultz WT (1977) Review of the genus *Rhabdopterus* (Coleoptera: Chrysomelidae) in America north of Mexico. Annals of the Entomological Society of America 70: 968–974.

[B486] Schultz WT (1980) A new species of *Nodonota* (Coleoptera: Chrysomelidae) with a review of the United States species. Annals of the Entomological Society of America 73: 200–203.

[B487] Schuster RO Marsh GA (1958) A study of the North American genus *Megarafonus* Casey (Coleoptera: Pselaphidae). The Pan-Pacific Entomologist 34: 187–194.

[B488] Schuster RO Grigarick AA (1960) A revision of the genus *Oropus* Casey (Coleoptera: Pselaphidae). Pacific Insects 2: 269–299.

[B489] Seevers CH (1951) A revision of the North American and European beetles of the subtribe Gyrophaenae (Aleocharinae, Bolitocharini). Fieldiana Zoology 32: 655–762.

[B490] Seevers CH Dybas H (1943) A synopsis of the Limulodidae (Coleoptera): a new family proposed for myrmecophiles of the subfamilies Limulodinae (Ptiliidae) and Cephaloplectinae (Staphylinidae). Annals of the Entomological Society of America 36: 546–586.

[B491] Selander RB (1955) The blister beetle genus *Linsleya* (Coleoptera, Meloidae). American Museum Novitates No. 1730: 30 pp.

[B492] Selander RB (1960) Bionomics, systematics and phylogeny of *Lytta*, a genus of blister beetles (Coleoptera: Meloidea). Illinois Biological Monographs No. 28: 295 pp.

[B493] Sen Gupta T (1967) A new subfamily of Languriidae (Coleoptera) based on four genera,with a key to the species of *Toramus*. Proceedings of the Royal Entomological Society of London, Series B, Taxonomy 36: 167–176. doi: 10.1111/j.1365-3113.1967.tb00532.x

[B494] Skelley PE (1997) A new species of *Dacne* Latreille from Dominican amber, with a key and checklist to the known species of *Dacne* (Erotylidae: Dacninae). Annales Zoologici 47: 49–53.

[B495] Skelley PE (1998) Revision of the genus *Ischyrus* Lacordaire (1842) of North and Central America (Coleoptera: Erotylidae: Tritominae). Occasional Papers of the Florida State Collection of Arthropods Vol. 9. i–viii + 134 pp.

[B496] Skiles DD (1985) New genera and species of Elaphidionine Cerambycidae (Coleoptera) from North America and the West Indies. The Coleopterists Bulletin 39: 305–320.

[B497] Sleeper EL (1955) A synopsis of the genus *Cophes* in the United States and Mexico (Coleoptera, Curculionidae). The Ohio Journal of Science 55: 188–191.

[B498] Sleeper EL (1956) A synopsis of the genus *Barinus* Casey in North America (Coleoptera Curculionidae). The Ohio Journal of Science 56: 76–86.

[B499] Sleeper EL (1957a) Notes on North American species of *Polydrusus* Germar (Coleoptera: Curculionidae, Brachyderinae). The Ohio Journal of Science 57: 129–134.

[B500] Sleeper EL (1957b) Notes on the genus *Macrorhoptus* LeConte (Coleoptera: Curculionidae, Anthonominae). The Ohio Journal of Science 57: 70–74.

[B501] Sleeper EL (1963) A study of the Zygopinae (Coleoptera: Curculionidae) of America north of Mexico, I. Bulletin of the Southern California Academy of Sciences 62: 209–220.

[B502] Ślipiński SA (1989) A review of the Passandridae (Coleoptera, Cucujoidea) of the world. 2. Genus *Catogenus* Westwood. Polskie Pismo Entomologiczne 59: 85–129.

[B503] Sloop KD (1937) A revision of the North American buprestid beetles belonging to the genus *Melanophila* (Coleoptera: Buprestidae). University of California Publications in Entomology No. 7: 19 pp.

[B504] Smetana A (1971) Revision of the tribe Quediini of America north of Mexico (Coleoptera: Staphylinidae). Memoirs of the Entomological Society of Canada No. 79: 303 pp.

[B505] Smetana A (1974) Revision of the genus *Cymbiodyta* Bed. (Coleoptera: Hydrophilidae). Memoirs of the Entomological Society of Canada No. 93: 113 pp.

[B506] Smetana A (1978) Revision of the subfamily Sphaeridiinae of America north of Mexico (Coleoptera: Hydrophilidae). Memoirs of the Entomological Society of Canada No. 105: 292 pp.

[B507] Smetana A (1980) Revision of the genus *Hydrochara* Berth. (Coleoptera: Hydrophilidae). Memoirs of the Entomological Society of Canada No. 111: 100 pp.

[B508] Smetana A (1981) *Ontholestes murinus* (Linné 1758) in North America (Coleoptera: Staphylinidae). The Coleopterists Bulletin 35: 125–126.

[B509] Smetana A (1982) Revision of the subfamily Xantholininae of America north of Mexico (Coleoptera: Staphylinidae). Memoirs of the Entomological Society of Canada No. 120: 389 pp.

[B510] Smetana A (1985a) Revision of the subfamily Helophorinae of the Nearctic Region (Coleoptera: Hydrophilidae). Memoirs of the Entomological Society of Canada No. 131: 154 pp.

[B511] Smetana A (1985b) Systematic position and review of *Deinopteroloma* Jansson, 1946, with descriptions of four new species (Coleoptera, Silphidae and Staphylinidae (Omaliinae)). Systematic Entomology 10: 471–499. doi: 10.1111/j.1365-3113.1985.tb00153.x

[B512] Smetana A (1988) Review of the family Hydrophilidae of Canada and Alaska (Coleoptera). Memoirs of the Entomological Society of Canada No. 142: 316 pp.

[B513] Smetana A (1995) Rove beetles of the subtribe Philonthina of America north of Mexico (Coleoptera, Staphylinidae) classification, phylogeny and taxonomic revision. Memoirs on Entomology, International No. 3: 946 pp.

[B514] Smith EH (1986) Revision of the genus *Phyllotreta* Chevrolat of America north of Mexico Part I. The maculate species (Coleoptera: Chrysomelidae, Alticinae). Fieldiana Zoology 28: 1–168.

[B515] Smith JW Balsbaugh EU Jr. (1984) A taxonomic revision of the Nearctic species of *Glyphonyx* (Coleoptera: Elateridae) with notes on *G. quadraticollis* Champion. North Dakota Insects Schafer-Post Series No. 16: 85 pp.

[B516] Smith SM Cognato AI (2010) Notes on *Scolytus fagi* Walsh 1867 with the designation of a neotype, distribution notes and a key to *Scolytus* Geoffroy of America east of the Mississippi River (Coleoptera, Curculionidae, Scolytinae, Scolytini). ZooKeys 56: 35–43. doi: 10.3897/zookeys.56.516 PMC3088332 21594170

[B517] Spilman TJ (1962) The New World genus *Centronopus*, with new generic synonymy and a new species (Coleoptera: Tenebrionidae). Transactions of the American Entomological Society 88: 1–19.

[B518] Springer CA Goodrich MA (1983) A revision of the family Byturidae (Coleoptera) for North America. The Coleopterists Bulletin 37: 183–192.

[B519] Staines CL (1986) A revision of the genus *Brachycoryna* (Coleoptera: Chrysomelidae: Hispinae). Insecta Mundi 1: 231–241.

[B520] Staines CL (2006) The hispine beetles of America north of Mexico (Chrysomelidae: Cassidinae). Virginia Museum of Natural History Special Publication No. 13: vi + 178 pp.

[B521] Stephan KH (1989) The Bothrideridae and Colydiidae of America north of Mexico (Coleoptera: Clavicornia and Heteromera). Occasional Papers of the Florida State Collection of Arthropods No. 6: 65 pp.

[B522] Stibick JNL (1976) A revision of the Hypnoidinae of the world (Col. Elateridae). Part I. Introduction, phylogeny, biogeography. The Hypnoidinae of North and South America. The genera *Berninelsonius* and *Ligmargus*. Eos, Revista Española de Entomologia 51: 143–223.

[B523] Stibick JNL (1978) A revision of the Hypnoidinae of the world (Col. Elateridae). Part II. The Hypnoidinae of North and South America. The genera *Ascoliocerus*, *Desolakerrus*, *Margaiostus*, *Hypolithus* and *Hypnoidus*. Eos, Revista Española de Entomologia 52: 309–386.

[B524] Stibick JNL (1991) North American Negastriinae (Coleoptera: Elateridae): the Negastriinae of the eastern United States and adjacent Canada. Insecta Mundi 4 [1990]: 99–131.

[B525] Stribling JB (1986) Revision of *Anchytarsus* (Coleoptera: Dryopoidea) and a key to the New World genera of Ptilodactylidae. Annals of the Entomological Society of America 79: 219–234.

[B526] Swift IP Ray AM (2010) Nomenclatural changes in North American *Phymatodes* Mulsant (Coleoptera: Cerambycidae). Zootaxa 2448: 35–52.

[B527] Tanner UM (1943) A study of the subtribe Hydronomi with a description of new species, (Curculionidae) study No. VI. The Great Basin Naturalist 4: 1–38.

[B528] Thomas MC (1984) A revision of the New World species of *Placonotus* Macleay (Coleoptera: Cucujidae: Laemophloeinae). Occasional Papers of the Florida State Collection of Arthropods No. 3: 28 pp.

[B529] Thomas MC (1988) A revision of the New World species of *Cryptolestes* Ganglbauer (Coleoptera: Cucujidae: Laemophloeinae). Insecta Mundi 2: 43–65.

[B530] Thomas MC (2004) A revision of *Pediacus* Shuckard (Coleoptera: Cucujidae) for America north of Mexico, with notes on other species. Insecta Mundi 17 [2003]: 157–177.

[B531] Triplehorn CA (1965) Revision of Diaperini of America north of Mexico with notes on extralimital species (Coleoptera: Tenebrionidae). Proceedings of the United States National Museum 117: 349–458. doi: 10.5479/si.00963801.117-3515.349

[B532] Triplehorn CA (1990) Review of the genus *Corticeus* (Coleoptera: Tenebrionidae) of America north of Mexico. Annals of the Entomological Society of America 83: 287–306.

[B533] Triplehorn CA Spilman TJ (1973) A review of *Strongilium* of America north of Mexico, with descriptions of two new species (Coleoptera, Tenebrionidae). Transactions of the American Entomological Society 99: 1–27.

[B534] Valentine BD (1960) The genera of the weevil family Anthribidae north of Mexico (Coleoptera). Transactions of the American Entomological Society 86: 41–85.

[B535] Vandenberg NJ (2002) The New World genus *Cycloneda* Crotch (Coleoptera: Coccinellidae: Coccinellini): historical review, new diagnosis, new generic and specific synonyms, and an improved key to North American species. Proceedings of the Entomological Society of Washington 104: 221–236.

[B536] Van Dyke EC (1928) The species of the genus *Lepyrus* Germ. (Coleoptera - Curculionidae) in North America. The Pan-Pacific Entomologist 5: 53–58.

[B537] Van Dyke EC (1933) A short review of *Dyslobus* LeConte, a genus of broad–nosed weevils of the subfamily Otiorhynchinae with descriptions of new species. The Pan-Pacific Entomologist 9: 31–47.

[B538] Vaurie P (1948) A review of the North American Languriidae. Bulletin of the American Museum of Natural History 92: 119–156.

[B539] Vaurie P (1951) Revision of the genus *Calandra* (formerly *Sphenophorus*) in the United States and Mexico (Coleoptera, Curculionidae). Bulletin of the American Museum of Natural History 98: 31–186.

[B540] Vaurie P (1955) A revision of the genus *Trox* in North America (Coleoptera: Scarabaeidae). Bulletin of the American Museum of Natural History 106: 1–89.

[B541] Vaurie P (1958) A revision of the genus *Diplotaxis* (Coleoptera: Scarabaeidae: Melolonthinae). Part I. Bulletin of the American Museum of Natural History 115: 265–396.

[B542] Vaurie P (1960) A revision of the genus *Diplotaxis* (Coleoptera: Scarabaeidae: Melolonthinae). Part 2. Bulletin of the American Museum of Natural History 120: 163–433.

[B543] Vaurie P (1981) Revision of *Rhodobaenus* Part 2. Species in North America (Canada to Panama) (Coleoptera, Curculionidae, Rhynchophorinae). Bulletin of the American Museum of Natural History 171: 119–198.

[B544] Wagner JA (1975) Review of the genera *Euplectus*, *Pycnoplectus*, *Leptoplectus*, and *Acolonia* (Coleoptera: Pselaphidae) including Nearctic species north of Mexico. Entomologica Americana 49: 125–207.

[B545] Wallis JB (1928) Revision of the genus *Odontaeus*, Dej. (Scarabaeidae: Coleoptera). The Canadian Entomologist 60: 119–128. doi: 10.4039/Ent60119b-5

[B546] Wallis JB (1933) Revision of the North American species (north of Mexico), of the genus *Haliplus*, Latreille. Transactions of the Royal Canadian Institute 19: 1–76.

[B547] Warner RE Negley FB (1976) The genus *Otiorhynchus* in America north of Mexico (Coleoptera: Curculionidae). Proceedings of the Entomological Society of Washington 78: 240–262.

[B548] Watrous L (1980) *Lathrobium (Tetartopeus)*: natural history, phylogeny and revision of the Nearctic species (Coleoptera: Staphylinidae). Systematic Entomology 5: 303–338. doi: 10.1111/j.1365-3113.1980.tb00418.x

[B549] Watson WY (1976) A review of the genus *Anatis* Mulsant (Coleoptera: Coccinellidae). The Canadian Entomologist 108: 935–944. doi: 10.4039/Ent108935-9

[B550] Wegrzynowicz P (1999) A revision of the genus *Colydium* Fabricius, 1792 (Coleoptera: Zopheridae: Colydiinae). Annales Zoologici, Warsaw 49: 265–328.

[B551] Wells SA (1991) Two new species of *Neohypdonus* (Coleoptera: Elateridae) from North America with a key to Nearctic species. Entomological News 102: 73–78.

[B552] Wells SA (1996) Studies on Nearctic *Negastrius* (Coleoptera: Elateridae). Great Basin Naturalist 56: 308–318.

[B553] Wells SA (2000) Two new species of *Horistonotus* Candeze (Coleoptera: Elateridae), new synonymies, and a key to the species of the United States and Canada. Proceedings of the Entomological Society of Washington 102: 412–420.

[B554] Werner FG (1958) A revision of the Nearctic species of *Tomoderus* (Coleoptera: Anthicidae). Psyche 64: 51–59. doi: 10.1155/1957/25706

[B555] Werner FG (1961) A revision of the genus *Vacusus* Casey (Coleoptera: Anthicidae). Annals of the Entomological Society of America 54: 798–809.

[B556] Werner FG (1962) A revision of the Nearctic species of *Sapintus* (Coleoptera: Anthicidae). Annals of the Entomological Society of America 55: 492–498.

[B557] Werner FG (1964) A revision of the North American species of *Anthicus*, s.str. (Coleoptera: Anthicidae). Miscellaneous Publications of the Entomological Society of America 4: 195–242.

[B558] Werner FG (1973) Revision of the Nearctic *Ischyropalpus* (Coleoptera: Anthicidae). Annals of the Entomological Society of America 66: 1055–1064.

[B559] Werner FG (1990) Revision of the Aderidae of eastern North America. Journal of the New York Entomological Society 98: 187–232.

[B560] Werner FG (1992) The Nearctic species of *Elonus* (Coleoptera: Aderidae). Psyche 99: 245–264. doi: 10.1155/1992/97483

[B561] Wheeler Q (1979) Slide mold beetles of the genus *Anisotoma* (Leiodidae): evolution and classification. Systematic Entomology 4: 251–309. doi: 10.1111/j.1365-3113.1979.tb00642.x

[B562] Wheeler Q Miller KB (2005) Slime-mold beetles of the genus *Agathidium* Panzer in North and Central America, Part I. Coleoptera: Leiodidae. Bulletin of the American Museum of Natural History No. 290: 95 pp.

[B563] White DS (1978) A revision of the Nearctic *Optioservus* (Coleoptera: Elmidae), with descriptions of new species. Systematic Entomology 3: 59–74. doi: 10.1111/j.1365-3113.1978.tb00390.x

[B564] White RE (1965) A revision of the genus *Tricorynus* of North America (Coleoptera: Anobiidae). Miscellaneous Publications of the Entomological Society of America 4: 285–368.

[B565] White RE (1966) Six new Anobiidae from North America with keys (Coleoptera). Proceedings of the Entomological Society of Washington 68: 228–236.

[B566] White RE (1968) A review of the genus *Cryptocephalus* in America north of Mexico (Chrysomelidae: Coleoptera). Bulletin of the United States National Museum No. 290: 124 pp.

[B567] White RE (1973a) New North American *Euvrilletta* and *Xyletinus* with keys to species (Coleoptera: Anobbiidae). Journal of the Washington Academy of Sciences 63: 76–80.

[B568] White RE (1973b) A new genus, two new species, and a species key to *Byrrhodes* (Coleoptera: Anobiidae). Proceedings of the Entomological Society of Washington 75: 48–54.

[B569] White RE (1975a) North American *Xestobium* (Anobiidae) with a new species. The Coleopterists Bulletin 29: 83–86.

[B570] White RE (1975b) Sixteen new Neotropical Anobiidae with a new genus and keys (Coleoptera). Proceedings of the Entomological Society of Washington 77: 169–188.

[B571] White RE (1976) Eight new North American species of Anobiidae with keys and notes (Coleoptera). Proceedings of the Entomological Society of Washington 78: 154–170.

[B572] White RE (1977) Ten new North American species of *Xyletinus* (Anobiidae: Coleoptera). Proceedings of the Entomological Society of Washington 79: 521–537.

[B573] White RE (1985) North American *Euvrilletta* (Coleoptera: Anobiidae) - Transferal of taxa from *Xyletinus*, two new species, and a key. The Coleopterists Bulletin 39: 185–193.

[B574] White RE (1993) A revision of the subfamily Criocerinae (Chrysomelidae) of North America north of Mexico. US Department of Agriculture Technical Bulletin No. 1805: v + 158 pp.

[B575] Whitehead DR (1972) Classification, phylogeny, and zoogeography of *Schizogenius* Putzeys (Coleoptera: Carabidae: Scaritini). Quaestiones Entomologicae 8: 131–348.

[B576] Whitehead DR (1979) Notes on *Apteromechus* Faust of America north of Mexico (Coleoptera: Curculionidae: Cryptorhynchinae). Proceedings of the Entomological Society of Washington 81: 230–233.

[B577] Whitehead DR Kingsolver JM (1975) Biosystematics of the North and Central American species of *Gibbobruchus* (Coleoptera: Bruchidae: Bruchinae). Transactions of the American Entomological Society 101: 167–225.

[B578] Wibmer GJ (1981) Revision of the New World weevil genus *Tyloderma* in America north of Mexico (Coleoptera: Curculionidae: Cryptorhynchinae). The Southwestern Entomologist Supplement No. 3: 95 pp.

[B579] Wilcox JA (1957) A revision of the North American species of *Paria* Lec. (Coleoptera: Chrysomelidae). Bulletin of the New York State Museum and Science Service No. 365: 45 pp.

[B580] Wilcox JA (1965) A synopsis of the North American Galerucinae (Coleoptera: Chrysomelidae). Bulletin of the New York State Museum and Science Service No. 400: 226 pp.

[B581] Wilcox JA (1972) A review of the North American chrysomeline leaf beetles (Coleoptera: Chrysomelidae). Bulletin of the New York State Museum and Science Service No. 421: 37 pp.

[B582] Will KW (1997) Review of the species of the subgenus *Megapangus* Casey (Coleoptera: Carabidae, Harpalini, *Harpalus* Latreille). The Coleopterists Bulletin 51: 43–51.

[B583] Will KW (1999) Systematics and zoogeography of the genus *Lophoglossus* Leconte (Coleoptera Carabidae Pterostichini). In: Zamotajlov A Sciaky R (Eds) Advances in carabidology. Papers dedicated to the memory of Prof. Dr. Oleg L. Kryzhanovskij. MUISO Publishers, Krasnodar, 259–276.

[B584] Wittmer W (1975) The genus *Phengodes* in the United States (Coleoptera: Phengodidae). The Coleopterists Bulletin 29: 231–250.

[B585] Wolfe GW Roughley RE (1990) A taxonomic, phylogenetic, and zoogeographic analysis of *Laccornis* Gozis (Coleoptera: Dytiscidae) with the description of Laccornini, a new tribe of Hydroporinae. Quaestiones Entomologicae 26: 273–354.

[B586] Wood SL (1982) The bark and ambrosia beetles of North and Central America (Coleoptera: Scotytidae), a taxonomic monograph. The Great Basin Naturalist Memoirs No. 6: 1359 pp.

[B587] Woodroffe GE Coombs CW (1961) A revision of the North American *Cryptophagus* Herbst (Coleoptera: Cryptophagidae). Miscellaneous Publications of the Entomological Society of America 2: 179–211.

[B588] Wooldridge DP (1977) New World Limnichinae III. A revision of *Limnichites* Casey (Coleoptera: Limnichidae). The Great Lakes Entomologist 10: 179–189.

[B589] Yensen E (1975) A revision of the North American species of *Trixagus* Kugelann (Coleoptera: Throscidae). Transactions of the American Entomological Society 101: 125–166.

[B590] Yensen E (1980) A new species of *Trixagus* from Panama and a key to New World *Trixagus* (Coleoptera: Throscidae). The Coleopterists Bulletin 34: 257–261.

[B591] Young DK (1975) A revision of the family Pyrochroidae (Coleoptera: Heteromera) for North America based on the larvae, pupae, and adults. Contributions of the American Entomological Institute (Ann Arbor) 11(3): 1–39.

[B592] Young FN (1953) Two new species of *Matus*, with a key to the known species and subspecies of the genus (Coleoptera: Dytiscidae). Annals of the Entomological Society of America 46: 49–55.

[B593] Young FN (1963) The Nearctic species of *Copelatus* Erichson (Coleoptera: Dytiscidae). Quarterly Journal of the Florida Academy of Sciences 26: 56–77.

[B594] Young FN (1979a) Water beetles of the genus *Suphisellus* Crotch in the Americas north of Colombia (Coleoptera: Noteridae). The Southwestern Naturalist 24: 409–429.

[B595] Young FN (1979b) A key to the Nearctic species of *Celina* with descriptions of new species (Coleoptera: Dytiscidae). Journal of the Kansas Entomological Society 52: 820–830. doi: 10.2307/3671297

[B596] Young FN (1985) A key to the American species of *Hydrocanthus* Say, with descriptions of new taxa (Coleoptera: Noteridae). Proceedings of the Academy of Natural Sciences of Philadelphia 137: 90–98.

[B597] Young RM (1988) A monograph of the genus *Polyphylla* Harris in America north of Mexico (Coleoptera: Scarabaeidae: Melolonthinae). Bulletin of the University of Nebraska State Museum No. 11(2): 115 pp.

[B598] Young RM (2002) A new *Cotalpa* Burmeister taken on post oak in eastern Texas with notes and a key to species in the genus (Scarabaeidae: Rutelinae). The Coleopterists Bulletin 56: 473–479. doi: 10.1649/0010-065X(2002)056[0473:ANCBTO]2.0.CO;2

[B599] Zerche L (2004) A revision of the genus *Aegialites* Mannerheim (Coleoptera: Salpingidae: Aegialitinae). Stuttgarter Beitraege zur Naturkunde Serie A (Biologie) 666: 1–116.

[B600] Zimmerman EC (1964) On the supposed North American Trachodinae (Coleoptera: Curculionidae). The Coleopterists Bulletin 18: 25–32.

[B601] Zimmerman JR (1970) A taxonomic revision of the aquatic beetle genus *Laccophilus* (Dytiscidae) of North America. Memoirs of the American Entomological Society No. 26: 275 pp.

[B602] Zimmerman JR (1981) A revision of the *Colymbetes* of North America. The Coleopterists Bulletin 35: 1–52.

[B603] Zimmerman JR (1985) The genus *Oreodytes* in North America (Coleoptera: Dytiscidae). Proceedings of the Philadelphia Academy of Sciences 137: 99–127.

[B604] Zimmerman JR Smith AH (1975) A survey of the *Deronectes* (Coleoptera: Dytiscidae) of Canada, the United States, and northern Mexico. Transactions of the American Entomological Society 101: 651–722.

[B605] Zimmerman JR Smith RL (1975) The genus *Rhantus* (Coleoptera: Dytiscidae) in North America. Part 1. General account of the species. Transactions of the American Entomological Society 101: 33–123.

